# The link between Wnt-related, stress-related, and circadian genes in the dermal fibroblasts of individuals with attention-deficit hyperactivity disorder

**DOI:** 10.1007/s00702-025-02986-0

**Published:** 2025-07-21

**Authors:** Denise Palm, Lukasz Smigielski, Adriana Uzoni, Oliver Tucha, Johannes Thome, Edna Grünblatt

**Affiliations:** 1 Department of Psychiatry and Psychotherapy, University Medical Centre Rostock, Rostock, Germany; 2 Department of Child and Adolescent Psychiatry and Psychotherapy, Psychiatric University Hospital Zurich, University of Zurich, Zurich, Switzerland; 3 Neuroscience Center Zurich, University of Zurich and the ETH Zurich, Zurich, Switzerland; 4 Zurich Center for Integrative Human Physiology, University of Zurich, Zurich, Switzerland

**Keywords:** ADHD, Gene expression, Circadian rhythms, Clock genes, Wnt signaling, *DKK1*

## Abstract

Attention-deficit/hyperactivity disorder (ADHD) has been associated with circadian rhythm disturbances, altered stress responses, and, in neural stem cells from ADHD patients, aberrant Wnt signaling. However, little is known about how these molecular pathways interact. This study aimed to investigate rhythmic expression of circadian, Wnt signaling, and stress-related genes in the context of ADHD. Human dermal fibroblasts were obtained via skin biopsy from participants diagnosed with ADHD (*n* = 13) and healthy controls (*n* = 13). Fibroblast cultures were synchronized using dexamethasone, with samples collected every 4 h over 28 h. Gene expression of Wnt signaling, stress-related, and circadian clock genes was quantified by qRT-PCR. Harmonic regression was applied to estimate rhythmicity (amplitude and phase), followed by mixed-effects modeling and likelihood ratio tests to assess between-group differences and gene–gene associations. Circular statistics (Rayleigh test, Watson two-sample test, circular correlations) were employed to test the uniformity and synchronicity of phase distributions. *BMAL1*, *CRY1*, *PER2*, *PER3*, and *DKK1* exhibited significant rhythmicity within each group. *DKK3* was rhythmic only in the ADHD group. Although between-group differences did not reach statistical significance, *BMAL1* and *CRY1* expression peaked later, while *PER2* and *PER3* expression peaked earlier in the ADHD group. Depending on data filtering, gene–gene rhythmicity associations included *CRY1–SIRT1*,* PER3–FOXO1*, and *CLOCK–CTNNB1* in ADHD subjects, as well as *CLOCK–DKK1* (ADHD) and *BMAL1–DKK1* in controls. The phase and amplitude of core clock genes were correlated with donors’ ADHD symptoms and subjective sleep measures. Our data indicate ADHD is associated with subtly altered circadian gene expression and distinct integration of Wnt signaling and stress-related pathways, supporting the hypothesis of broader molecular dysregulation underlying ADHD.

**Supplementary Information:**

The online version contains supplementary material available at 10.1007/s00702-025-02986-0.

## Introduction

Attention-deficit hyperactivity disorder (ADHD) is a neurodevelopmental condition characterized by patterns of inattention, hyperactivity, and impulsivity (Walitza et al. 2014). It persists into adulthood in over 60% of cases, with significant societal and economic ramifications (Song et al. 2021). Recent research has begun to unravel the complex biological underpinnings of ADHD, emphasizing the interplay between genetic, environmental, and neurobiological factors. In this context, the Wnt signaling pathway has garnered attention for its role in key cellular processes such as proliferation, differentiation, and synaptic plasticity (Yde Ohki et al. 2020). Alterations in the function of Wnt ligands and proteins can lead to anomalies in embryonic development and has been linked to various neurodevelopmental disorders, including autism spectrum disorder (ASD), schizophrenia, and neurodegenerative conditions, such as Alzheimer’s disease (AD) (Inestrosa et al. 2012; Zou and Salinas 2014; Grünblatt et al. 2023; Bou Najm et al. 2025). Wnt plays a crucial role in maintaining the regulation of free β-catenin pools (encoded by the *CTNNB1* gene) through the “canonical” pathways reliant on β-catenin. Conversely, the “non-canonical” pathway operates independently of β-catenin (Shi 2024). Recent data corroborate the involvement of canonical Wnt signaling in the etiopathology of ADHD (Yde Ohki et al. 2020).

Concurrently, stress-related genes have been identified as important contributors to ADHD pathophysiology (van der Meer et al. 2016). The HPA axis interacts with neurodevelopmental pathways, further complicating the disorder’s etiology (Yde Ohki et al. 2020). Moreover, mitochondrial dysfunction and the resulting increase in reactive oxygen species (ROS) have also been implicated in the exacerbation of ADHD symptoms, suggesting an important role of oxidative stress in the disorder’s progression (Yde Ohki et al. 2020). Forkhead box protein 1 (FOXO1) and sirtuin 1 (SIRT1) are central regulators of oxidative stress (Hori et al. 2013), influencing not only mitochondrial function, but also neuroinflammation and neuronal survival (Mormone et al. 2023). Particularly noteworthy are the molecular processes by which FOXO1 and SIRT1 modulate Wnt signaling in response to oxidative stress. SIRT1 interacts with FOXO1, facilitating its deacetylation and enhancing its transcriptional activity, including regulation of stress-response genes like GADD45 and Mn-SOD (Mormone et al. 2023). Under stress conditions (e.g., inflammation), ROS mediate the Wnt/β-catenin pathway by inhibiting SIRT1, resulting in increased FOXO acetylation. Acetylated FOXO then translocates to the nucleus, where it interacts with β-catenin to activate transcription of genes involved in oxidative stress resistance, inflammation, and apoptosis (Almeida et al. 2007; Li et al. 2021a). Cicek et al. (2020) demonstrated markedly reduced serum SIRT1 levels in individuals with ADHD, which was correlated with symptom severity and cognitive function (Uzun Cicek et al. 2020).

Moreover, the intricate relationship between Wnt signaling and stress-related genes extends beyond ADHD, suggesting its broader implications for neurodevelopmental disorders (Zwamborn et al. 2018; Sahoo et al. 2011; Khaliulin et al. 2025). For instance, recent evidence indicates that dysregulation of Wnt pathways can exacerbate stress responses, potentially leading to altered circadian rhythms, which are often disrupted in individuals with ADHD (Matsu-Ura et al. 2018). This intersection underscores how environmental stressors influence behavioral outcomes and interact with genetic predispositions through molecular mechanisms involving Wnt signaling. As researchers continue to explore these connections, it is becoming increasingly evident that a deeper understanding of these pathways could unveil novel therapeutic targets for mitigating symptoms associated with ADHD and related disorders.

Furthermore, circadian genes, which regulate the body’s internal clock and sleep-wake cycles, have been linked to ADHD symptoms, suggesting a potential disruption of biological rhythms. Specifically, the circadian rhythm genes *PER2*, *CRY1*, *BMAL1*, and *CLOCK* showed altered synchrony and expression in ADHD human-derived dermal fibroblasts (HDFs) synchronized by dexamethasone (Coogan et al. 2019). The Wnt signaling pathway is also associated with genes regulating circadian rhythms. Reduced levels of PER3 negatively affect stemness and elevate BMAL1 expression, which in turn promote β-catenin and activation of the Wnt/β-catenin pathway (Li et al. 2021b). Notably, BMAL1 knockdown in osteoblasts suppressed GSK3β/β-catenin communication, reducing β-catenin expression and GSK-3β phosphorylation at serine 9. Conversely, GSK3β inhibition by TDZD-8—but not by WNT3a or SKL2001—ameliorated the differentiation impairments caused by BMAL1 knockdown in osteoblasts (Li et al. 2023). Indeed, the Wnt pathway itself appears to follow a circadian rhythm, influencing the cell cycle, as well as stem cell proliferation and differentiation (Matsu-Ura et al. 2018).

On the other hand, the interaction between SIRT1 and FOXO transcription factors plays an important role in regulating circadian rhythms (Duffy et al. 2020; Jenwitheesuk et al. 2017; Draijer et al. 2023) and may also be implicated in ADHD. SIRT1, a key regulator of cellular metabolism and energy homeostasis, influences the expression of clock-controlled genes essential for maintaining circadian rhythms (Osum and Serakinci 2020; Mormone et al. 2023). FOXO factors, involved in stress responses and longevity, interact with SIRT1 to regulate gene expression related to attention and impulse control (Jenwitheesuk et al. 2017; Greer and Brunet 2005). Disruptions in circadian rhythm regulation in ADHD can lead to sleep disturbances and attention deficits, underscoring the importance of these pathways in the disorder (Bondopadhyay et al. 2021; Coogan et al. 2019; Imeraj et al. 2012).

Drawing from the above insights, elucidating the interplay between the Wnt pathway, SIRT1, FOXO, and circadian rhythms opens new avenues for advancing our understanding of ADHD-related molecular mechanisms. Modulating these signaling pathways may offer a promising strategy for managing ADHD symptoms more effectively. This study aims to elucidate the relationships among Wnt signaling (*LRP6*, *CTNBB1*, *DKK1*, *DKK3*), stress-related gene expression (*SIRT1*, *FOXO1*), and circadian gene regulation (*CLOCK*, *BMAL1*, *CRY1*, *PER1*, *PER2*, *PER3*) in HDFs from individuals with ADHD. By investigating these connections, we aim to clarify molecular mechanisms underlying ADHD and identify potential biomarkers for diagnostic and therapeutic use.

## Methods

### Participant selection criteria

The study was conducted in compliance with the ethical standards outlined in the Declaration of Helsinki. The research obtained ethical approval from the Ethical Review Committee of Rostock University (registration number, A2013-159), and all participants provided informed written consent. Individuals diagnosed with ADHD (non-medicated; *n* = 13) and those without any neuropsychiatric disorders were recruited through the Department of Psychiatry and Psychotherapy at the University Medical Centre Rostock. All participants diagnosed with ADHD were assessed by experienced psychiatrists. The control group (*n* = 13) comprised persons acquainted with the study participants. Controls were matched for sex and age and had no history of childhood or adult ADHD. Participants displaying symptoms indicative of other comorbid conditions or severe mental symptoms, as well as shift workers, were excluded. The severity of ADHD symptoms was evaluated using the Wender Utah Rating Scale (WURS-25) (Ward 1993) and Conners’ Adult ADHD Rating Scales (CAARS) (Christiansen et al. 2011), along with symptom evaluation according to the criteria outlined in the DSM-IV (Association 2000) and ICD-10 (Organization 2004). The ADHD diagnosis was confirmed using the following psychometric assessments: SKID I and II (Structured Clinical Interview) (Wittchen et al. 1997; Fydrich et al. 1997), DIVA 2.0 (Structured Diagnostic Interview) (Ramos-Quiroga et al. 2019), and Pittsburgh Sleep Quality Index (PSQI) (Buysse et al. 1989). The Multiple Choice Word Test (MWT) was employed to evaluate the IQ of both the control group and those diagnosed with ADHD (Lehrl et al. 1995). The participants’ chronotype was assessed using the German version of the Morning-Eveningness Questionnaire (D-MEQ) (Griefahn et al. 2001). Human dermal fibroblasts (HDFs) were collected from skin biopsies of the dorsal forearm of individuals diagnosed with ADHD and a control group. Only adult participants capable of providing informed consent were included.

### Tissue isolation and fibroblast cell culture

The isolation and culture of HDFs were conducted using a previously described procedure (Faltraco et al. 2021c; Coogan et al. 2019; Palm et al. 2021; Takashima 2001). In brief, HDFs were cultured at 37 °C under 5% CO_2_ using Dulbecco’s Modified Eagle Medium (DMEM) (Gibco, Thermo Fisher, Cambridge, UK) supplemented with 1 mg/ml Liberase TM (Roche, Mannheim, Germany). The medium contained 100 units/ml penicillin, 100 µg/ml streptomycin (Gibco, Thermo Fisher), and 10% fetal bovine serum (FBS) (Gibco, Thermo Fisher).

### HDF culture and synchronization via dexamethasone

HDF line cultures were evaluated at eight time points, i.e., initially and then every 4 h thereafter for 28 h. For each HDF line evaluated at the eight intervals, we created eight culture flask replicas and synchronized them with 100 nM dexamethasone (Sigma-Aldrich, Taufkirchen, Germany) for 30 min, following the confluency of the corresponding HDF cell culture from each participant. In our review article (Faltraco et al. 2023), we compared various synchronization methods and concluded that dexamethasone is a well-established and widely used protocol for synchronizing fibroblasts ex vivo. This method has also been validated in several foundational studies demonstrating that dexamethasone induces robust circadian rhythms in cells cultured in medium containing 5–20% serum (Balsalobre et al. 2000; Nagoshi et al. 2004; Izumo et al. 2006). Samples were collected at 4-hour intervals post-synchronization over a 28-hour period (designated as the “zeitgeber” period ZT0 to ZT28) in solution D (comprising 4.5 M guanidinium thiocyanate, 0.5% sodium-*N*-lauryl sarcosine, 25 mM tri-sodium citrate, and 0.1 M beta-mercaptoethanol). The collected samples were thereafter preserved at a temperature of -80 °C until further isolation.

### Expression of WNT-related, stress-related, and circadian genes

Total RNA was extracted and purified from HDFs using the RNeasy Plus Mini Kit (Qiagen, Hilden, Germany). RNA purity and RNA concentration were assessed spectrophotometrically using the Multiskan Go instrument (Thermo Fisher Scientific, Waltham, MA, USA). RNA was measured by its absorbance at 260 and 280 nm (A260 and A280, respectively). The concentration was calculated using the A260 value and a conversion factor based on a previously determined standard curve.

The Superscript III First-Strand Synthesis System (Invitrogen, Darmstadt, Germany) was utilized to reverse transcribe RNA into cDNA. The real-time quantitative reverse transcriptase polymerase chain reaction (qRT-PCR) method was used to quantify the expression of Wnt- and stress-related genes (*CTNNB1*, *DKK1*, *DKK3*, *LRP6*, *SIRT1*, *FOXO1*), circadian genes (*CLOCK*, *BMAL1*, *PER1*, *PER2*, *PER3*, *CRY1*), and reference genes (*Rpl13A*, *Rpl19A*, *GAPDH*) using the CFX Connect™ Real-Time PCR Detection System (Bio-Rad Laboratories, Feldkirchen, Germany). Table S1 (Supplement Information) presents the oligonucleotide sequences and PCR conditions. The qRT-PCR assay was performed using 96-well 0.1-ml thin-wall PCR plates (Applied Biosystems, Waltham, MA, USA), adhering to the previously outlined methodology. Primer efficiency, ranging from 95.0 to 100.0%, was assessed using LinRegPCR v 11.0 (Untergasser et al. 2021). The expression levels of target genes were normalized to the geometric mean of the reference genes (*Rpl13A*, *Rpl19A*, and *GAPDH*) from the same sample (Mane et al. 2008). Each gene was quantified using at least two technical replicates per cell line. If the standard deviation between replicates exceeded 0.5 cycles, two additional replicates were performed and included in the analysis. The mean Ct value from the technical replicates was used for expression analysis. Gene expression was analyzed using the delta-delta Ct technique (Coogan et al. 2019; Faltraco et al. 2021c; Palm et al. 2021).

$$ \Delta {\text{Ct }} = {\text{ Ct }}\left( {{\text{time point}}} \right){-}{\text{Ct }}\left( {{\text{Max of all 8 time points}}} \right) $$

$$\:\varDelta\:\varDelta\:Ct=\:\frac{\varDelta\:Ct\:\left(gene\:of\:interest\right)}{\varDelta\:Ct\:\left(reference\:genes\right)}$$

$$ {\text{Fold Change }} = {\text{ 2}}^{{ - \Delta \Delta {\mathrm{Ct}}}} $$

The values were normalized to the corresponding individual averages.

### Statistical methods

All statistical analyses were performed in R (R Project for Statistical Computing, version 4.4.2). To obtain an overall view of periodicity, we first applied harmonic regression analyses, followed by more specific circular statistical analyses.

### Harmonic regression analyses and visualizations

Gene expression data were first analyzed for significant rhythmicity by assessing the parameters of phase (i.e., timing of peak expression) and amplitude (i.e., strength of rhythmic oscillations). At the group level, a harmonic regression model with mixed effects fitted in the ‘lme4’ package was used to test for 24-hour oscillations in gene expression incorporating sine and cosine transformations, separately, for the control and ADHD groups. To explore the variance structure, principal component analysis (PCA) was conducted, leading to the exclusion of one time point for one sample based on PC plot inspection (Supplementary Figure S1). In the within-group rhythmicity analysis, two models were compared: the *null model*, which included only a random intercept for each individual, and the *full model*, which additionally incorporated cosine and sine transformations of the time variables. The likelihood ratio test (LRT) was used to compare both models, and a gene was considered rhythmic if the LRT was significant at a threshold of *p* < 0.05. Furthermore, between-group rhythmicity was tested using linear mixed-effects models with sine- and cosine-transformed variables and the same LRT approach. The *null model* assumed shared rhythmicity across groups, fixed-effect sine- and cosine-transformed variables, and a random intercept for each participant. The *alternative model* allowed group-specific rhythmicity by including an interaction with group. We further visualized the phase–amplitude relationship of rhythmic genes at the individual level (i.e., for each participant) using polar coordinate plots. In the next step, to investigate whether there was synchronicity or regularity between clock genes and Wnt signaling/stress-related genes, linear regressions were performed for both phase and amplitude. These analyses were conducted using both (i) all available samples (i.e., unfiltered) and (ii) a more stringent subset of at least three data points in which both genes in each pair exhibited significant rhythmicity (filtered; *p* < 0.05, based on the LRT and harmonic regression fits).

### Circular statistics and visualizations

Circular statistics, in which values represent points in a circular cycle (e.g., 24 h or 360°), were conducted using the ‘circular’ R package. The phase values were converted to radians. Rayleigh tests were used to investigate the uniformity of a circular distribution of points for each gene in the ADHD and control groups. To test whether there was a significant difference between the two groups in their central tendency phase differences, the Watson two-sample test was applied. Circular correlations between clock genes and WNT signaling/stress-related genes were calculated separately for the control and ADHD groups. Participant-level circadian phase values (in hours) were converted to radians and analyzed using the *cor.circular* function in the R package ‘circular,’ according to the method described by Jammalamadaka and Sarma (Jammalamadaka and Sarma 1988).

As an exploratory analysis, we also performed Spearman’s correlations between the phase and amplitude of gene expression and the available clinical (CAARS) and sleep (PSQI) scores in ADHD patients.

## Results

### Demographic data

Among the HDFs obtained via skin biopsy from 13 healthy controls (HC; 4 men, 9 women) and 13 ADHD individuals (5 men, 7 women) no statistically significant between-group variation was observed regarding age (HC, 47.15 ± 18.82 years; ADHD, 45.92 ± 15.98 years; n.s.), BMI (HC, 26.90 ± 6.36 kg/m²; ADHD, 26.04 ± 4.33 kg/m²; n.s.), MWT scores as a proxy for IQ (HC, 111.77 ± 6.36; ADHD, 107.46 ± 12.86; n.s.), or the overall D-MEQ score (HC, 61.23 ± 8.81; ADHD, 50.00 ± 12.94; n.s). However, the D-MEQ scores indicated that those subjects diagnosed with ADHD had a significant and moderate preference for evenings relative to the control group (χ^2^(3,23) = 32.23, *p* < 0.001). Specifically, 46.2% of HC subjects had a neutral preference, whereas 38.5% displayed a moderate preference for the morning. Furthermore, 15.4% of HC subjects indicated a distinct morning preference. In addition, 30.8% of participants with ADHD exhibited a moderate preference for mornings, 53.8% remained neutral, and 15.4% preferred evenings (7.7% moderately and 7.7% definitively).

As expected, marked differences were observed between groups for ADHD symptom severity, as shown by WURS-25 scores (HC, 5.08 ± 7.54; ADHD, 40.92 ± 13.27; t(24) = 8.464, *p* < 0.001). Table 1 presents the detailed participant characteristics.

**Table 1:** Participant characteristics

Demographic data	Healthy controls *n* = 13	ADHD *n* = 13
Age	47.15 ± 18.82 years	45.92 ± 15.98 years
Female	9 (69.2%)	7 (58.3%)
BMI	26.90 ± 6.36 kg/m^2^	26.04 ± 4.33 kg/m^2^
IQ-Score	111.77 ± 6.96	107.46 ± 12.86
D-MEQ	61.23 ± 8.81	50.00 ± 12.94
WURS-k-Score	5.08 ± 7.54	40.92 ± 13.27***
Chronotype	Neutral: 46.2% Moderate morning: 38.5% Definitive morning: 15.4%	Neutral: 53.8% Moderate morning: 30.8% Definitive evening: 7.7% Moderate evening: 7.7% ^**+**^

***, *t*(24) = 8.464, *p* < 0.001 (*t*-test); ^+^, χ^2^(3, 23) = 32.23, *p* < 0.001 (χ^2^ test). Abbreviations: BMI, body mass index; D-MEQ:, Morning-Eveningness Questionnaire; WURS, Wender Utah Rating Scale

### Harmonic regression analyses

**Fig. 1:**
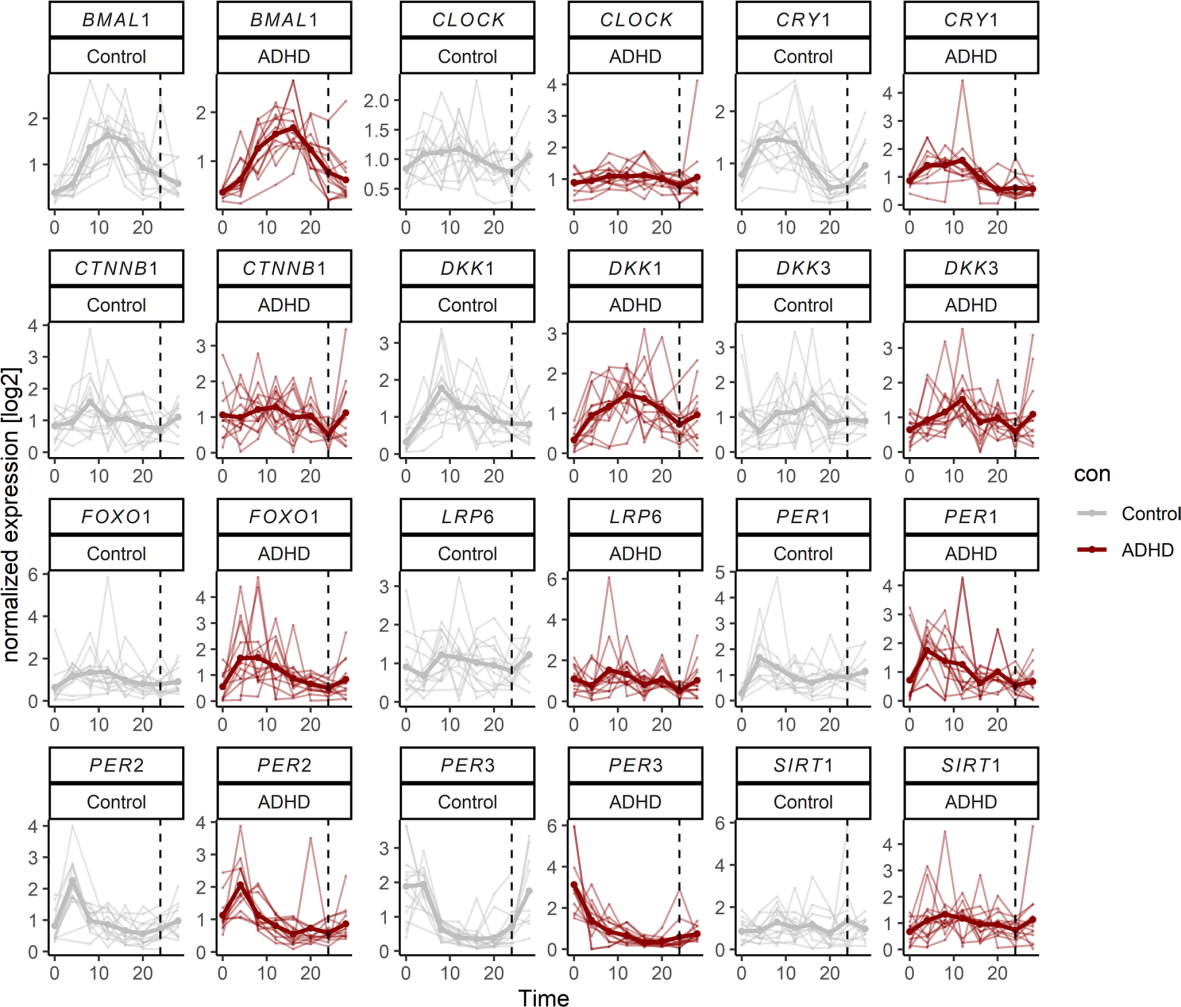
Gene expression time courses in the control and ADHD groups. Normalized gene expression of circadian rhythm-, Wnt-, and stress-related genes was quantified via qRT-PCR of human dermal fibroblasts (HDFs) from individuals with ADHD (*n* = 13) and matched healthy controls (*n* = 13) over a 28-hour period following synchronization by 100 nM dexamethasone treatment. Data points represent averaged qRT-PCR values from technical replicates per cell line (minimum *n* = 2)

The gene expression profiles over time are visualized for each group in Fig. 1.

Several genes, including *BMAL1*, *CRY1*, *PER2*, *PER3*, and *DKK1*, exhibited significant rhythmicity in both groups (*p* < 0.05), suggesting that these genes maintain their oscillatory expression patterns regardless of ADHD status. Notably, *DKK3* and *FOXO1* showed significant effects only in the ADHD group (*p* < 0.05). In contrast, *CLOCK*, *CTNNB1*, *LRP6*, *PER1*, and *SIRT1* did not exhibit significant rhythmicity (*p* > 0.05) in either group. Among the rhythmic genes, *PER3* in particular exhibited a clearly lower amplitude in ADHD subjects (1.155) compared to control subjects (1.524), indicative of weaker oscillation. Data also suggested slight phase shifts for *BMAL1*, *DKK1*, and *CRY1* (peaking later in ADHD, i.e., 14.03 vs. 13.07, 12.54 vs. 10.97, 9.89 vs. 8.99, respectively), as well as *PER2* and *PER3* (shifting toward an earlier phase in ADHD, i.e., 5.36 vs. 5.74, 1.87 vs. 3.18, respectively). The rhythmicity statistics are provided in Table 2 and in Figure S2, which presents the differences in the LRT. Individual-level rhythmicity from this analysis is depicted as polar plots in Supplementary Figure S3.

**Table 2:** Statistical summary of gene expression rhythmicity in the control and ADHD groups

	Control Group			ADHD Group		
Gene	Amplitude	Phase	*p*	Amplitude	Phase	*p*
*BMAL1*	1.188	13.071	**< 0.001**	1.203	14.026	**< 0.001**
*CLOCK*	0.380	9.879	0.286	0.255	11.910	0.185
*CTNNB1*	0.574	9.355	0.208	0.440	10.588	0.848
*CRY1*	0.987	8.999	**< 0.001**	0.949	9.892	**< 0.001**
*DKK1*	0.947	10.968	**< 0.001**	0.843	12.537	**< 0.001**
*DKK3*	0.427	14.551	0.636	0.681	10.812	**0.032**
*FOXO1*	0.708	9.988	0.195	1.147	8.969	**0.002**
*LRP6*	0.328	10.957	0.271	0.547	10.536	0.893
*PER1*	0.731	6.949	0.075	0.722	8.507	0.289
*PER2*	0.944	5.737	**< 0.001**	0.862	5.363	**0.002**
*PER3*	1.524	3.183	**< 0.001**	1.155	1.870	**0.003**
*SIRT1*	0.182	9.803	0.126	0.554	9.446	0.743

The *p*-values of statistically significant differences in the likelihood ratio test (LRT), indicative of significant rhythmicity, are shown in bold

No statistically significant differences were found in the between-group analysis (Supplementary Table S2).

### Rhythmicity between clock genes and Wnt signaling/stress-related genes

In the unfiltered analyses for phase (*n* = 13 per group), the following gene pairs reached statistical significance in the control group: *CLOCK‒LRP6* (*R* = -0.69 (inverse), *R*² = 0.47, *p* = 0.009), *PER1‒FOXO1* (*R* = 0.63, *R*² = 0.39, *p* = 0.022), and *PER1‒DKK3* (*R* = 0.58, *R*² = 0.34, *p* = 0.037). In the ADHD group, significant phase associations were found for *CRY1‒SIRT1* (*R* = 0.74, *R*² = 0.55, *p* = 0.0039), *CLOCK‒LRP6* (*R* = 0.73, *R*² = 0.53, *p* = 0.005), *CLOCK‒CTNNB1* (*R* = 0.69, *R*² = 0.48, *p* = 0.009), *PER3‒SIRT1* (*R* = -0.63 (inverse), *R*² = 0.39, *p* = 0.022), and *PER3‒FOXO1* (*R* = -0.59 (inverse), *R*² = 0.35, *p* = 0.032). In the filtered analyses, only the *CLOCK–DKK1* pair was significant in the ADHD group (*R* = -0.99, *R*² = 0.99, *p* = 0.020, *n* = 3). In the unfiltered analysis for amplitude (*n* = 13 per group), significant negative associations were found in the control group between *BMAL1* and *LRP6* (*R* = -0.64, *R*² = 0.42, *p* = 0.017) and between *BMAL1* and *FOXO1* (*R* = -0.65, *R*² = 0.42, *p* = 0.017), as well as in the ADHD group between *PER3* and *DKK1* (*R* = -0.67, *R*² = 0.45, *p* = 0.012). In the filtered analysis (*n* = 4), a positive association was observed between *BMAL1* and *DKK1* in the control group (*R* = 0.96, *R*² = 0.92, *p* = 0.040). No multiple testing corrections were applied in these analyses. See Supplementary Tables S3–S6 for detailed results.

### Circular statistics

**Fig. 2:**
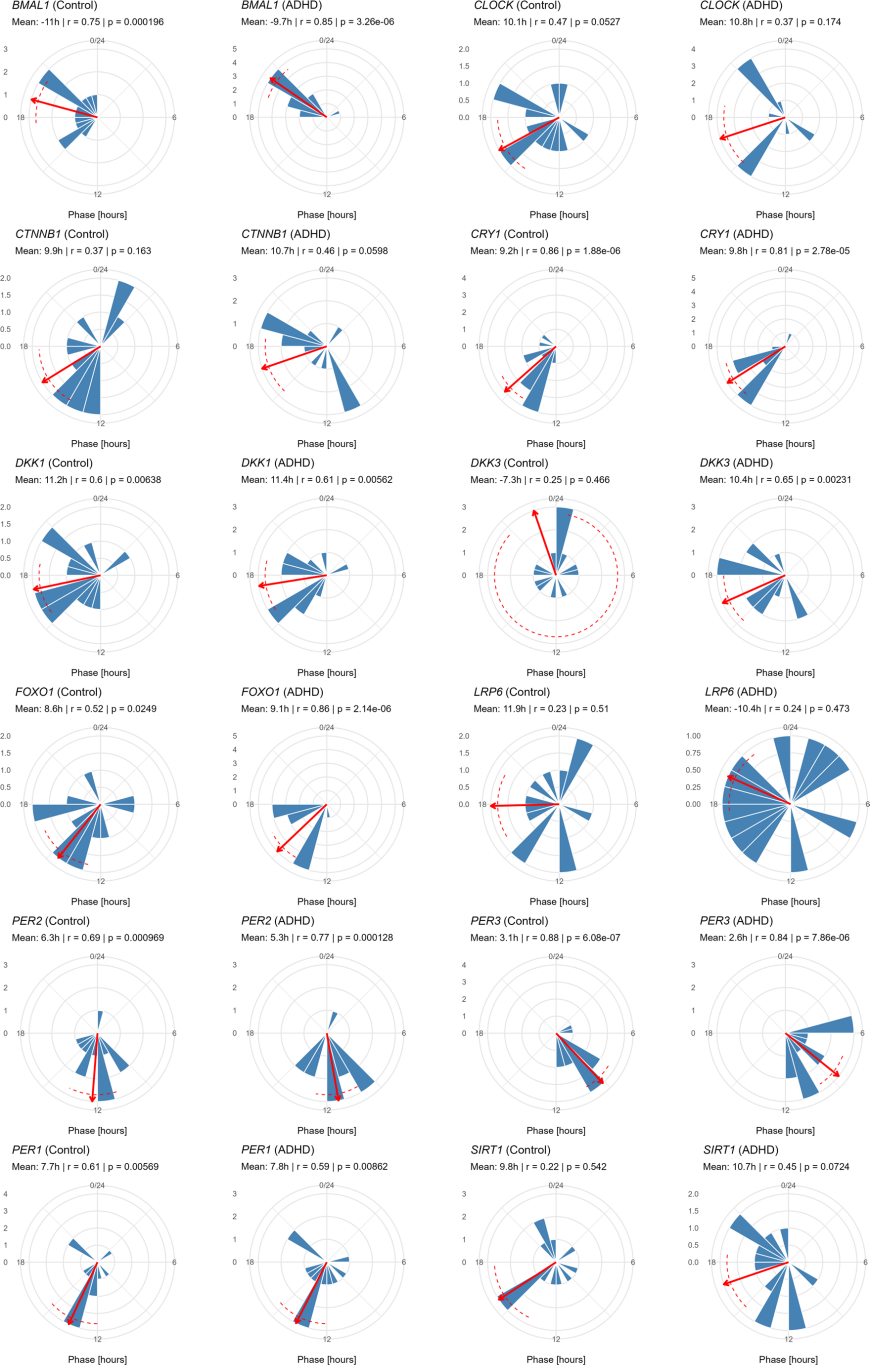
Rayleigh plots of phase clustering across genes. Each plot displays the distribution of peak expression phases across participants for the indicated gene. Arrows indicate the mean direction (phase), and dashed arcs represent 95% confidence intervals of the mean. Rayleigh test *p*-values indicate the significance of the non-uniformity of phase distributions (i.e., evidence of rhythmicity), while the *r* value (vector strength) quantifies the degree of clustering around the mean (0 = random, 1 = tightly clustered). Plots are shown separately for control (*n* = 13) and ADHD (*n* = 13) groups. Data points represent averaged qRT-PCR values from technical replicates per cell line (minimum *n* = 2)

Rayleigh tests confirmed significant phase clustering for several genes, with results being largely in agreement with the harmonic regression estimates (Fig. 2, Supplementary Table S7). In both the control and ADHD groups, the core circadian genes *BMAL1*, *CRY1*, *PER2*, and *PER3* exhibited strong and consistent rhythmicity (Rayleigh *p*-values < 0.001 and vector strengths > 0.75). *DKK1* was also rhythmic in both groups (*p* = 0.006 and *r* ≈ 0.61). *PER1* showed significant clustering in both groups (*p* ≈ 0.006–0.009 and *r* ≈ 0.59–0.61), whereas it was not found to be rhythmic in the harmonic regression. *FOXO1* showed significant rhythmicity in both groups (ADHD, *p* < 0.001, *r* = 0.86; control, *p* = 0.025, *r* = 0.52), whereas harmonic regression indicated significance only in the ADHD group (*p* = 0.002). *DKK3* showed significant clustering in ADHD subjects only (*p* = 0.002, *r* = 0.65), supporting the harmonic regression finding. *CLOCK*, *CTNNB1*, *LRP6*, and *SIRT1* were not rhythmic in either group, paralleling the harmonic regression findings.

Watson two-sample tests comparing phase distributions between control and ADHD groups for 12 genes showed no statistically significant effects (Table 3). Circular–circular correlation analyses (supplementary Table S8) revealed several significant associations between the phase angles of circadian genes and WNT signaling/stress-related genes for the ADHD group (*CLOCK–CTNNB1*, ρ = 2.17, *p* = 0.029; *CRY1–SIRT1*, ρ = 3.12, *p* = 0.002; *PER2‒SIRT1*, ρ = 2.12, *p* = 0.034; *PER2–FOXO1*, ρ = 2.73, *p* = 0.006; *PER3‒FOXO1*, ρ = 2.09, *p* = 0.036; *PER1–SIRT1*, ρ = − 2.31, *p* = 0.021) and the control group (*CRY1–FOXO1*, ρ = − 2.15, *p* = 0.031).

**Table 3:** Watson two-sample test results for group differences in circadian phase (control vs. ADHD)

Gene	*n* controls	*n* ADHD	W statistics	Significance	*p*
*BMAL1*	13	13	0.118	No	> 0.10
*CLOCK*	13	13	0.061	No	> 0.10
*CTNNB1*	13	13	0.098	No	> 0.10
*CRY1*	13	13	0.078	No	> 0.10
*DKK1*	13	13	0.059	No	> 0.10
*DKK3*	13	13	0.182	No	0.05 < *p* < 0.10
*FOXO1*	13	13	0.137	No	> 0.10
*LRP6*	13	13	0.031	No	> 0.10
*PER2*	13	13	0.059	No	> 0.10
*PER3*	13	13	0.077	No	> 0.10
*PER1*	13	13	0.042	No	> 0.10
*SIRT1*	13	13	0.066	No	> 0.10

*n* = number of individuals per group; W = Watson U² test statistic; *p* = *p*-value from Watson test; Significance threshold, *p* < 0.05. Gray rows indicate genes with significant phase distributions (*p* < 0.05) in both groups based on the group-level Rayleigh test

Results of the exploratory correlational analyses are presented in Supplementary Table S9 and Supplementary Figures S4 and S5. For CAARS, significant correlations were observed for the amplitudes of BMAL1 (*r* = 0.87, *p* = 0.005) and PER1 (*r* = 0.80, *p* = 0.014), as well as the phase of PER3 (*r* = 0.72, *p* = 0.037) (*n* = 9). For PSQI, only the phase of PER3 was significantly associated (*r* = 0.74, *p* = 0.022; *n* = 9). However, none of these associations remained significant after False Discovery Rate (FDR) correction.

## Discussion

This study examined whether Wnt- and stress-related genes synchronize and are co-expressed with circadian genes and whether such expression is disrupted in ADHD, potentially contributing to the disorder’s neurodevelopmental phenotype. Several methodological approaches, including harmonic regression and circular statistical modeling, were applied to maximize the insights gained from our experimental data. Corroborating prior findings (Coogan et al. 2019; Faltraco et al. 2021a, b, c), dexamethasone-induced synchronization in HDFs from individuals with ADHD and control subjects elicited rhythmic expression of key circadian genes, including *BMAL1*, *CRY1*, *PER2*, and *PER3*. No statistically significant differences between groups were found in the analyses. However, a shift in the phase of gene expression was observed in individuals with ADHD; *BMAL1* and *CYR1* peaked later than in control HDFs, whereas *PER2* and *PER3* peaked earlier in ADHD HDFs. *CLOCK* and *BMAL1* encode components of a heterodimeric complex that binds to promotor elements of *CYR1* as well as *PER1*, *PER2*, and *PER3* to initiate their expression. *CRY1*, *PER1*, *PER2*, and *PER3* are part of a negative feedback-loop that inhibits the CLOCK–BMAL1 complex and thereby suppresses their own expression (Lee et al. 2001; Kume 1999; Shearman et al. 2000). The observed slight temporal shifts in ADHD relative to controls may have affected the negative feedback loop, potentially exacerbating circadian dysregulation in patients with ADHD. These phase shifts in core clock genes may have cascading effects on the transcriptional timing of downstream targets. Genes involved in neural plasticity, such as BDNF and its receptor TrkB, are regulated by circadian clock components, linking circadian disruption to critical neurodevelopmental processes, including synaptic plasticity and neuronal survival (Liu et al. 2015; Ali et al. 2019). Dopamine-related genes, including those encoding enzymes for synthesis and transport, are also under circadian control (Akkaya et al. 2023). Moreover, clock genes are involved in monoamine degradation (Mogavero et al. 2018). Interestingly, in our exploratory correlational analyses with clinical and subjective sleep measures, the BMAL1–PER1–PER3 axis, components of the core and secondary circadian feedback loops, emerged as particularly relevant and may reflect or contribute to dysregulation of sleep and attention in ADHD. Although these mechanisms are not yet fully elucidated, molecular asynchrony may contribute to the behavioral phenotype observed in ADHD.

The observed expression of Wnt pathway components in our study reinforces the putative connection between circadian rhythms and neurodevelopmental pathways in ADHD. In prostate cancer stem cells, *PER3* has been shown to enhance the expression of *BMAL1*, resulting in the phosphorylation of β-catenin and subsequent activation of the Wnt/β-catenin pathway (Li et al. 2021b). The Wnt pathway has been linked to ADHD at the genetic (Grünblatt et al. 2019; Yde Ohki et al. 2020), molecular, and cellular levels, as also evidenced by research on neurons derived from induced pluripotent stem cells (iPSCs) (Walter et al. 2024). The Wnt pathway has been shown to exhibit circadian rhythmicity, affecting the cell cycle, as well as the proliferation and differentiation of stem cells (Matsu-Ura et al. 2018). In support of the above findings, *DKK1*, an antagonist of Wnt signaling, exhibited rhythmic expression in both ADHD and control groups in our study, with a delayed phase shift observed in ADHD cells, similar to *BMAL1* and *CRY1*. Furthermore, *DKK3* exhibited rhythmic expression exclusively in ADHD cells. Alterations in the oscillatory patterns of *DKK1* and *DKK3* in ADHD may affect neurogenesis, neural patterning, and development (Fukusumi et al. 2015; Seib et al. 2013); however, further investigation is required to evaluate this hypothesis.

Stressors may further influence gene expressions patterns, particularly those related to the circadian clock. Existing evidence indicates that disruptions in circadian rhythms contribute to anxiety and other stress-related disorders, which are often comorbid with ADHD (Landgraf et al. 2014). *FOXO1* is a transcription factor that translocates to the nucleus and is activated in response to oxidative stress and inflammatory processes (Hori et al. 2013). Translocation into the nucleus can activate Wnt signaling through an alternative mechanism (Almeida et al. 2007; Li et al. 2021a). Notably, in our study, *FOXO1* exhibited circadian rhythmicity, particularly in HDFs from ADHD subjects, which may indirectly indicate stress-induced activation. Recent evidence has shown heightened oxidative stress and modifications in inflammatory processes in iPSC-derived forebrain cortical neurons from individuals with ADHD (Walter et al. 2025).

Several gene‒gene rhythmicity associations between clock genes and Wnt signaling/stress-related genes were observed in the ADHD group. Specifically, *CRY1–SIRT1*, *PER3–FOXO1*, and *CLOCK–CTNNB1* were identified by both linear regressions for phase and circular‒circular correlation analyses. Putative co-regulation between *CRY1* (a circadian repressor) and *SIRT1* (a member of the sirtuin family engaged in metabolic and stress responses) may reflect redox- or stress-related circadian adaptations in ADHD. *SIRT1* is involved in regulating cellular stress responses and modulates circadian clock function by deacetylating core clock components (Nakahata et al. 2008). Our findings may indicate that both genes are regulated through a shared negative feedback loop. Notably, previous studies have reported reduced SIRT1 levels in serum from ADHD patients, which were also correlated with symptom severity (Uzun Cicek et al. 2020). Another gene pair of interest is *PER3–FOXO1*, in which *FOXO1* is typically activated by stress and *PER3* is associated with sleep and circadian regulation. Their functional coupling may reflect coordinated regulation or reciprocal control. *PER* and *CRY1* are components of the negative feedback loop, while *FOXO1* and SIRT1 engage in mutual interaction (Mormone et al. 2023). Finally, rhythmic co-expression of *CLOCK* and *CTNNB1* (which encodes β-catenin and regulates cellular homeostasis) suggests circadian gating of Wnt signaling, which may have implications for time-dependent regulation of neurodevelopmental processes. Notably, recent findings indicate that *CTNNB1* and its various polymorphisms may play a significant role in neurodevelopmental disorders by disrupting the Wnt signaling pathway, thereby affecting synaptic plasticity, apoptosis, and neurogenesis (Zhuang et al. 2023).

While the findings from the filtered data should be interpreted with caution owing to the small sample size of the present study, they offer added value by focusing on a stringent subset in which both genes showed robust rhythmicity based on harmonic regression fits. Notably, in controls, *BMAL1*–*DKK1* exhibited associated rhythmicity, whereas in ADHD, it was *CLOCK*–*DKK1*. *BMAL1* and *CLOCK* form a heterodimeric complex that binds to transcription factor-binding sites (Menet et al. 2014), suggesting that the timing of *DKK1* expression may regulate the entire circadian loop. The delay in *DKK1* expression observed in HDFs from ADHD patients may also have influenced the regulation of *CLOCK*, potentially explaining the synchrony between *DKK1* and *CLOCK* in ADHD cells. Taken together, the cross-gene results from this study suggest that, alongside alterations in circadian genes in ADHD patient cells, stress- and Wnt-related genes likely interact, providing preliminary evidence for altered rhythmic coordination.

While HDFs provide a valuable ex vivo model for assessing molecular and circadian processes, gene expression patterns differ across cell types. Thus, the observed effects may not fully reflect molecular mechanisms in neuronal cells, which are distinctively specialized in function, exhibit unique synaptic properties, and engage in activity-dependent signaling pathways absent in fibroblasts (Cerneckis et al. 2024; Xu et al. 2020). Future studies employing iPSC-derived neuronal cell types could offer deeper insight into the neurobiological relevance of our findings. Moreover, such systems would permit temporal manipulation of gene expression, for example, via siRNA-mediated knockdown or CRISPR interference/activation, to test the causality of gene–gene interactions such as the FOXO1–PER3 pair. Time-resolved gene silencing or overexpression under synchronized conditions could help determine whether one gene directly regulates the oscillation of another, thereby strengthening the mechanistic links proposed in this study.

Although the case–control HDFs used in the study were meticulously selected and matched, it is important to acknowledge the limitations of the present work. The sample size limited the power to detect subtle variation in rhythmicity, and the statistical tests were not corrected for multiple comparisons, qualifying these analyses as exploratory. Therefore, replication in a larger and independent cohort is essential. Unfortunately, no data were available on clinical treatment responsiveness for the ADHD patients. Our previous work (Coogan et al. 2019), however, showed that *PER2* and *CRY1* expression differed between unmedicated and medicated ADHD individuals or controls, while *CLOCK* expression was specifically altered in those currently on medication, reflecting possible modulatory effects of medication. Furthermore, potential epigenetic modifications resulting from environmental influences or pharmaceutical agents cannot be precluded when using HDF cells. In this context, the use of iPSC-derived neurons may reveal a more obvious gene expression profile following dexamethasone induction, presuming that reprogramming of somatic cells eliminates pre-existing epigenetic markers. Finally, gene expression may not accurately reflect alterations in protein levels; therefore, evaluating protein expression and their phosphorylated states (i.e., activated/inactivated forms) will be crucial for reaching definitive conclusions.

## Conclusions

In summary, the findings from this study underscore the importance of integrating circadian biology into ADHD research and treatment. The still not fully understood interactions between circadian genes, Wnt signaling, and stress responses may be utilized to identify potential biomarkers and novel therapeutic targets. These may include pharmacological interventions, lifestyle modifications, and behavioral therapies aimed at restoring the dysregulated molecular rhythms associated with ADHD. Some evidence suggests that the efficacy of atomoxetine, a non-stimulant medication used to treat ADHD, may be partially mediated through its influence on circadian rhythms (O’Keeffe et al. 2012). Continued research is necessary to clarify the mechanisms linking circadian dysregulation, stress responses, and Wnt signaling in the context of ADHD.

## Supplementary Information

Below is the link to the electronic supplementary material.

Supplementary file1 (PDF 1981 KB)

## Data Availability

Data and material are available upon reasonable request. The analysis code is also available upon reasonable request.

## Abbreviations

AD: Alzheimer’s disease
ADHD: Attention-deficit hyperactivity disorder
ASD: Autism spectrum disorder
CAARS: Conners’ adult ADHD rating scales
DMEM: Dulbecco’s modified eagle medium
D-MEQ: Morning-eveningness questionnaire
DSM-IV: Diagnostic and statistical manual of mental disorders, fourth edition
FDR: False discovery rate
FOXO1: Forkhead box protein 1
HC: Healthy control
HDFs: Human dermal fibroblasts
ICD-10: International classification of diseases, 10th revision
iPSCs: Induced pluripotent stem cells
MWT: Multiple choice word test
PCA: Principal component analysis
PSQI: Pittsburgh sleep quality index
qRT-PCR: Quantitative real-time reverse transcription polymerase chain reaction
ROS: Reactive oxygen species
SIRT1: Sirtuin 1
SKID: Structured clinical interview
WURS-25: Wender Utah rating scale

## References

[CR1] Akkaya C, Karadag M, Hangul Z, Sahin E, Isbilen E (2023) Evaluation of the regulatory role of circadian rhythm related long Non-Coding RNAs in ADHD etiogenesis. J Atten Disord 27(2):201–213 36254757 10.1177/10870547221130113

[CR2] Ali AA, Schwarz-Herzke B, Mir S, Sahlender B, Victor M, Görg B, Schmuck M, Dach K, Fritsche E, Kremer A (2019) Deficiency of the clock gene Bmal1 affects neural progenitor cell migration. Brain Struct Function 224:373–386 10.1007/s00429-018-1775-1 PMC6373387 30341743

[CR3] Almeida M, Han L, Martin-Millan M, O’Brien CA, Manolagas SC (2007) Oxidative stress antagonizes Wnt signaling in osteoblast precursors by diverting β-catenin from T cell factor-to forkhead box O-mediated transcription. J Biol Chem 282(37):27298–27305 17623658 10.1074/jbc.M702811200

[CR4] Association AP (2000) Diagnostic and statistical manual of mental disorders IV-TR Washington. American Psychiatric Association, DC

[CR5] Balsalobre A, Brown SA, Marcacci L, Tronche F, Kellendonk C, Reichardt HM, Schutz G, Schibler U (2000) Resetting of circadian time in peripheral tissues by glucocorticoid signaling. Science 289(5488):2344–2347 11009419 10.1126/science.289.5488.2344

[CR6] Bondopadhyay U, Diaz-Orueta U, Coogan AN (2021) The role of the circadian system in attention deficit hyperactivity disorder. Circadian Clock Brain Health Disease:113–127 10.1007/978-3-030-81147-1_7 34773229

[CR7] Bou Najm D, Alame S, Takash Chamoun W (2025) Unraveling the role of Wnt signaling pathway in the pathogenesis of autism spectrum disorder (ASD): A systematic review. Mol Neurobiol 62(4):4971–4992. 10.1007/s12035-024-04558-x 39489840 10.1007/s12035-024-04558-x

[CR8] Buysse DJ, Reynolds CF, Monk TH, Berman SR, Kupfer DJ (1989) The Pittsburgh sleep quality index: a new instrument for psychiatric practice and research. Psychiatry Res 28(2):193–213. 10.1016/0165-1781(89)90047-4 2748771 10.1016/0165-1781(89)90047-4

[CR9] Cerneckis J, Cai H, Shi Y (2024) Induced pluripotent stem cells (iPSCs): molecular mechanisms of induction and applications. Signal Transduct Target Therapy 9(1):112 10.1038/s41392-024-01809-0 PMC11053163 38670977

[CR10] Christiansen H, Kis B, Hirsch O, Philipsen A, Henneck M, Panczuk A, Pietrowsky R, Hebebrand J, Schimmelmann B (2011) German validation of the Conners adult ADHD rating Scales–self-report (CAARS-S) I: factor structure and normative data. Eur Psychiatry 26(2):100–107 20619613 10.1016/j.eurpsy.2009.12.024

[CR11] Coogan AN, Schenk M, Palm D, Uzoni A, Grube J, Tsang AH, Kolbe I, McGowan NM, Wandschneider R, Colla M, Oster H, Thome J, Faltraco F (2019) Impact of adult attention deficit hyperactivity disorder and medication status on sleep/wake behavior and molecular circadian rhythms. Neuropsychopharmacology 44(7):1198–1206. 10.1038/s41386-019-0327-6 30758328 10.1038/s41386-019-0327-6 PMC6785110

[CR12] Draijer S, Timmerman R, Pannekeet J, van Harten A, Farshadi EA, Kemmer J, van Gilst D, Chaves I, Hoekman MF (2023) FoxO3 modulates circadian rhythms in neural stem cells. Int J Mol Sci 24(17):13662 37686468 10.3390/ijms241713662 PMC10563086

[CR13] Duffy T, Bekki H, Lotz MK (2020) Genome-wide occupancy profiling reveals critical roles of FOXO1 in regulating extracellular matrix and circadian rhythm genes in human chondrocytes. Arthritis Rheumatol 72(9):1514–1523 32281255 10.1002/art.41284 PMC7813518

[CR14] Faltraco F, Palm D, Coogan A, Uzoni A, Duwe I, Simon F, Tucha O, Thome J (2021a) Remdesivir shifts circadian rhythmicity to eveningness; similar to the most prevalent chronotype in ADHD. J Neural Transm 128:1159–1168 34273024 10.1007/s00702-021-02375-3 PMC8285716

[CR15] Faltraco F, Palm D, Uzoni A, Borchert L, Simon F, Tucha O, Thome J (2021b) Dopamine adjusts the circadian gene expression of Per2 and Per3 in human dermal fibroblasts from ADHD patients. J Neural Transm 128:1135–1145 34275001 10.1007/s00702-021-02374-4 PMC8295132

[CR16] Faltraco F, Palm D, Uzoni A, Simon F, Tucha O, Thome J (2021c) Atomoxetine and circadian gene expression in human dermal fibroblasts from study participants with a diagnosis of attention-deficit hyperactivity disorder. J Neural Transm (Vienna). 10.1007/s00702-021-02373-5 34273025 10.1007/s00702-021-02373-5 PMC8295110

[CR17] Faltraco F, Uzoni A, Shevchuk L, Thome J, Palm D (2023) Synchronization of fibroblasts ex vivo in psychopharmacology. Pharmacopsychiatry 56(03):101–107 32340062 10.1055/a-1151-4947

[CR18] Fukusumi Y, Meier F, Götz S, Matheus F, Irmler M, Beckervordersandforth R, Faus-Kessler T, Minina E, Rauser B, Zhang J (2015) Dickkopf 3 promotes the differentiation of a rostrolateral midbrain dopaminergic neuronal subset in vivo and from pluripotent stem cells in vitro in the mouse. J Neurosci 35(39):13385–13401 26424886 10.1523/JNEUROSCI.1722-15.2015 PMC6605475

[CR19] Fydrich T, Renneberg B, Schmitz B, Wittchen H-U SKID II. Strukturiertes Klinisches Interview für DSM-IV, Achse II: Persönlichkeitsstörungen. Interviewheft. Eine deutschspeachige, erw. Bearb. d. amerikanischen Originalversion d. SKID-II von: MB First, Spitzer RL, Gibbon M (1997) JBW Williams, L. Benjamin,(Version 3/96)

[CR20] Greer EL, Brunet A (2005) FOXO transcription factors at the interface between longevity and tumor suppression. Oncogene 24(50):7410–7425. 10.1038/sj.onc.1209086 16288288 10.1038/sj.onc.1209086

[CR21] Griefahn B, Künemund C, Bröde P, Mehnert P (2001) Zur validität der Deutschen übersetzung des morningness-eveningness‐questionnaires von Horne und östberg: the validity of a German version of the morningness‐eveningness‐questionnaire developed by Horne and Östberg. Somnologie 5(2):71–80

[CR23] Grünblatt E, Nemoda Z, Werling AM, Roth A, Angyal N, Tarnok Z, Thomsen H, Peters T, Hinney A, Hebebrand J (2019) The involvement of the canonical Wnt-signaling receptor LRP5 and LRP6 gene variants with ADHD and sexual dimorphism: association study and meta‐analysis. Am J Med Genet Part B: Neuropsychiatric Genet 180(6):365–376 10.1002/ajmg.b.32695 PMC6767385 30474181

[CR22] Grünblatt E, Homolak J, Babic Perhoc A, Davor V, Knezovic A, Osmanovic Barilar J, Riederer P, Walitza S, Tackenberg C, Salkovic-Petrisic M (2023) From attention-deficit hyperactivity disorder to sporadic alzheimer’s disease—Wnt/mTOR pathways hypothesis. Front NeuroSci 17:1104985 36875654 10.3389/fnins.2023.1104985 PMC9978448

[CR24] Hori YS, Kuno A, Hosoda R, Horio Y (2013) Regulation of FOXOs and p53 by SIRT1 modulators under oxidative stress. PLoS ONE 8(9):e73875. 10.1371/journal.pone.0073875 24040102 10.1371/journal.pone.0073875 PMC3770600

[CR25] Imeraj L, Sonuga-Barke E, Antrop I, Roeyers H, Wiersema R, Bal S, Deboutte D (2012) Altered circadian profiles in attention-deficit/hyperactivity disorder: an integrative review and theoretical framework for future studies. Neurosci Biobehav Rev 36(8):1897–1919. 10.1016/j.neubiorev.2012.04.007 22575380 10.1016/j.neubiorev.2012.04.007

[CR26] Inestrosa NC, Montecinos-Oliva C, Fuenzalida M (2012) Wnt signaling: role in alzheimer disease and schizophrenia. J Neuroimmune Pharmacol 7(4):788–807. 10.1007/s11481-012-9417-5 23160851 10.1007/s11481-012-9417-5

[CR27] Izumo M, Sato TR, Straume M, Johnson CH (2006) Quantitative analyses of circadian gene expression in mammalian cell cultures. PLoS Comput Biol 2(10):e136 17040123 10.1371/journal.pcbi.0020136 PMC1599765

[CR28] Jammalamadaka SR, Sarma YR (1988) A correlation coefficient for angular variables. Stat Theory Data Anal II:349–364

[CR29] Jenwitheesuk A, Boontem P, Wongchitrat P, Tocharus J, Mukda S, Govitrapong P (2017) Melatonin regulates the aging mouse hippocampal homeostasis via the sirtuin1-FOXO1 pathway. EXCLI J 16:340 28507478 10.17179/excli2016-852 PMC5427465

[CR30] Khaliulin I, Hamoudi W, Amal H (2025) The multifaceted role of mitochondria in autism spectrum disorder. Mol Psychiatry 30(2):629–650. 10.1038/s41380-024-02725-z 39223276 10.1038/s41380-024-02725-z PMC11753362

[CR31] Kume KZ, Sriram MJ, Shearman S, Weaver LP, Jin DR, Maywood X, Hastings ES, Reppert MH, SM (1999) mCRY1 and mCRY2 are essential components of the negative limb of the circadian clock feedback loop. Cell 98(2):193–205 10428031 10.1016/s0092-8674(00)81014-4

[CR32] Landgraf D, McCarthy MJ, Welsh DK (2014) Circadian clock and stress interactions in the molecular biology of psychiatric disorders. Curr Psychiatry Rep 16:1–11 10.1007/s11920-014-0483-7 25135782

[CR33] Lee C, Etchegaray JP, Cagampang FR, Loudon AS, Reppert SM (2001) Posttranslational mechanisms regulate the mammalian circadian clock. Cell 107(7):855–867. 10.1016/s0092-8674(01)00610-9 11779462 10.1016/s0092-8674(01)00610-9

[CR34] Lehrl S, Triebig G, Fischer B (1995) Multiple choice vocabulary test MWT as a valid and short test to estimate premorbid intelligence. Acta Neurol Scand 91(5):335–345 7639062 10.1111/j.1600-0404.1995.tb07018.x

[CR35] Li C, Sheng M, Lin Y, Xu D, Tian Y, Zhan Y, Jiang L, Coito AJ, Busuttil RW, Farmer DG, Kupiec-Weglinski JW, Ke B (2021a) Functional crosstalk between myeloid Foxo1-β-catenin axis and Hedgehog/Gli1 signaling in oxidative stress response. Cell Death Differ 28(5):1705–1719. 10.1038/s41418-020-00695-7 33288903 10.1038/s41418-020-00695-7 PMC8167164

[CR37] Li Q, Xia D, Wang Z, Liu B, Zhang J, Peng P, Tang Q, Dong J, Guo J, Kuang D, Chen W, Mao J, Li Q, Chen X (2021b) Circadian rhythm gene PER3 negatively regulates stemness of prostate Cancer stem cells via WNT/beta-Catenin signaling in tumor microenvironment. Front Cell Dev Biol 9:656981. 10.3389/fcell.2021.656981 33816508 10.3389/fcell.2021.656981 PMC8012816

[CR36] Li H, Meng H, Xu M, Gao X, Sun X, Jin X, Sun H (2023) BMAL1 regulates osteoblast differentiation through mTOR/GSK3β/β-catenin pathway. J Mol Endocrinol 70 (4) 10.1530/JME-22-0181 36942818

[CR38] Liu D-Y, Shen X-M, Yuan F-F, Guo O-Y, Zhong Y, Chen J-G, Zhu L-Q, Wu J (2015) The physiology of BDNF and its relationship with ADHD. Mol Neurobiol 52:1467–1476 25354496 10.1007/s12035-014-8956-6

[CR39] Mane VP, Heuer MA, Hillyer P, Navarro MB, Rabin RL (2008) Systematic method for determining an ideal housekeeping gene for real-time PCR analysis. J Biomol Tech 19(5):342–347 19183798 PMC2628067

[CR40] Matsu-Ura T, Moore SR, Hong CI (2018) WNT takes two to tango: molecular links between the circadian clock and the cell cycle in adult stem cells. J Biol Rhythms 33(1):5–14. 10.1177/0748730417745913 29277155 10.1177/0748730417745913 PMC6324728

[CR41] Menet JS, Pescatore S, Rosbash M (2014) CLOCK: BMAL1 is a pioneer-like transcription factor. Genes Dev 28(1):8–13 24395244 10.1101/gad.228536.113 PMC3894415

[CR42] Mogavero F, Jager A, Glennon JC (2018) Clock genes, ADHD and aggression. Neurosci Biobehavioral Reviews 91:51–68 10.1016/j.neubiorev.2016.11.002 27836462

[CR43] Mormone E, Iorio EL, Abate L, Rodolfo C (2023) Sirtuins and redox signaling interplay in neurogenesis, neurodegenerative diseases, and neural cell reprogramming. Front Neurosci 17:1073689. 10.3389/fnins.2023.1073689 36816109 10.3389/fnins.2023.1073689 PMC9929468

[CR44] Nagoshi E, Saini C, Bauer C, Laroche T, Naef F, Schibler U (2004) Circadian gene expression in individual fibroblasts: cell-autonomous and self-sustained oscillators pass time to daughter cells. Cell 119(5):693–705 15550250 10.1016/j.cell.2004.11.015

[CR45] Nakahata Y, Kaluzova M, Grimaldi B, Sahar S, Hirayama J, Chen D, Guarente LP, Sassone-Corsi P (2008) The NAD+-dependent deacetylase SIRT1 modulates CLOCK-mediated chromatin remodeling and circadian control. Cell 134(2):329–340 18662547 10.1016/j.cell.2008.07.002 PMC3526943

[CR46] O’Keeffe S, Thome J, Coogan A (2012) The noradrenaline reuptake inhibitor Atomoxetine phase-shifts the circadian clock in mice. Neuroscience 201:219–230 22119060 10.1016/j.neuroscience.2011.11.002

[CR47] Organization WH (2004) International statistical classification of diseases and related health problems: alphabetical index, vol 3. World Health Organization

[CR48] Osum M, Serakinci N (2020) Impact of circadian disruption on health; SIRT1 and telomeres. DNA Repair (Amst) 96:102993. 10.1016/j.dnarep.2020.102993 33038659 10.1016/j.dnarep.2020.102993

[CR49] Palm D, Uzoni A, Simon F, Tucha O, Thome J, Faltraco F (2021) Norepinephrine influences the circadian clock in human dermal fibroblasts from study participants with a diagnosis of attention-deficit hyperactivity disorder. J Neural Transm (Vienna). 10.1007/s00702-021-02376-2 34275002 10.1007/s00702-021-02376-2 PMC8295072

[CR50] Ramos-Quiroga JA, Nasillo V, Richarte V, Corrales M, Palma F, Ibáñez P, Michelsen M, Van de Glind G, Casas M, Kooij JS (2019) Criteria and concurrent validity of DIVA 2.0: a semi-structured diagnostic interview for adult ADHD. J Atten Disord 23(10):1126–1135 27125994 10.1177/1087054716646451

[CR51] Sahoo PK, Murawala P, Sawale PT, Sahoo MR, Tripathi MM, Gaikwad SR, Seshadri V, Joseph J (2011) Wnt signalling antagonizes stress granule assembly through a Dishevelled-dependent mechanism. Biology Open 1(2):109–119 23213403 10.1242/bio.2011023 PMC3507204

[CR52] Seib DR, Corsini NS, Ellwanger K, Plaas C, Mateos A, Pitzer C, Niehrs C, Celikel T, Martin-Villalba A (2013) Loss of Dickkopf-1 restores neurogenesis in old age and counteracts cognitive decline. Cell Stem Cell 12(2):204–214 23395445 10.1016/j.stem.2012.11.010

[CR53] Shearman LP, Sriram S, Weaver DR, Maywood ES, Chaves I, Zheng B, Kume K, Lee CC, van der Horst GT, Hastings MH, Reppert SM (2000) Interacting molecular loops in the mammalian circadian clock. Science 288(5468):1013–1019 10807566 10.1126/science.288.5468.1013

[CR54] Shi DL (2024) Canonical and Non-Canonical Wnt signaling generates molecular and cellular asymmetries to Establish embryonic axes. J Dev Biol 12(3). 10.3390/jdb12030020 10.3390/jdb12030020 PMC11348223 39189260

[CR55] Song P, Zha M, Yang Q, Zhang Y, Li X, Rudan I (2021) The prevalence of adult attention-deficit hyperactivity disorder: A global systematic review and meta-analysis. J Global Health 11:04009 10.7189/jogh.11.04009 PMC7916320 33692893

[CR56] Takashima A (2001) Establishment of fibroblast cultures. Curr Protoc Cell Biol Chap. 2:Unit 2 1. 10.1002/0471143030.cb0201s00 10.1002/0471143030.cb0201s00 18228346

[CR57] Untergasser A, Ruijter JM, Benes V, van den Hoff MJB (2021) Web-based linregpcr: application for the visualization and analysis of (RT)-qPCR amplification and melting data. BMC Bioinformatics 22(1):398. 10.1186/s12859-021-04306-1 34433408 10.1186/s12859-021-04306-1 PMC8386043

[CR58] Uzun Cicek A, Mercan Isik C, Bakir S, Ulger D, Sari SA, Bakir D, Cam S (2020) Evidence supporting the role of telomerase, MMP-9, and SIRT1 in attention-deficit/hyperactivity disorder (ADHD). J Neural Transm (Vienna) 127(10):1409–1418. 10.1007/s00702-020-02231-w 32691156 10.1007/s00702-020-02231-w

[CR59] van der Meer D, Hoekstra PJ, Bralten J, van Donkelaar M, Heslenfeld DJ, Oosterlaan J, Faraone SV, Franke B, Buitelaar JK, Hartman CA (2016) Interplay between stress response genes associated with attention-deficit hyperactivity disorder and brain volume. Genes Brain Behav 15(7):627–636. 10.1111/gbb.12307 27391809 10.1111/gbb.12307

[CR60] Walitza S, Romanos M, Greenhill L, Banaschewski T (2014) Attention-deficit/hyperactivity disorders. Psychiatric Drugs Child Adolescents: Basic Pharmacol Practical Appl:369–381

[CR61] Walter NM, Ohki CMY, Rickli M, Smigielski L, Walitza S, Grünblatt E (2024) An investigation on the alterations in Wnt signaling in ADHD across developmental stages. Neurosci Appl 3:104070 40656072 10.1016/j.nsa.2024.104070 PMC12244180

[CR62] Walter NM, Ohki CMY, Smigielski L, Walitza S, Grünblatt E (2025) Investigating the impact of omega-3 fatty acids on oxidative stress and pro-inflammatory cytokine release in iPSC-derived forebrain cortical neurons from ADHD patients. J Psychiatr Res 182:257–269 39826376 10.1016/j.jpsychires.2025.01.020

[CR63] Ward MF (1993) The Wender Utah rating scale: an aid in the retrospective diagnosis of childhood attention deficit hyperactivity disorder. Am J Psychiatry 150:885–885 8494063 10.1176/ajp.150.6.885

[CR64] Wittchen H-U, Fydrich T, Zaudig M (1997) SKID: strukturiertes klinisches interview für DSM-IV; achse I und II. achse I: Psychische störungen. SKID-I. Hogrefe

[CR65] Xu Z, Su S, Zhou S, Yang W, Deng X, Sun Y, Li L, Li Y (2020) How to reprogram human fibroblasts to neurons. Cell Bioscience 10:1–25 33062254 10.1186/s13578-020-00476-2 PMC7549215

[CR66] Yde Ohki CM, Grossmann L, Alber E, Dwivedi T, Berger G, Werling AM, Walitza S, Grünblatt E (2020) The stress–Wnt-signaling axis: a hypothesis for attention-deficit hyperactivity disorder and therapy approaches. Translational Psychiatry 10(1):315 32948744 10.1038/s41398-020-00999-9 PMC7501308

[CR67] Zhuang W, Ye T, Wang W, Song W, Tan T (2023) CTNNB1 in neurodevelopmental disorders. Front Psychiatry 14:1143328 37009120 10.3389/fpsyt.2023.1143328 PMC10061110

[CR68] Zou Y, Salinas P (2014) Introduction: Wnt signaling mechanisms in development and disease. Dev Neurobiol 74(8):757–758. 10.1002/dneu.22192 24845245 10.1002/dneu.22192

[CR69] Zwamborn RA, Snijders C, An N, Thomson A, Rutten BP, de Nijs L (2018) Wnt signaling in the hippocampus in relation to neurogenesis, neuroplasticity, stress and epigenetics. Prog Mol Biol Transl Sci 158:129–157 30072051 10.1016/bs.pmbts.2018.04.005

