# Supplementary material for: The link between Wnt-related, stress-related, and circadian genes in the dermal fibroblasts of individuals with attention-deficit hyperactivity disorder
Source: J Neural Transm (Vienna). 2025 Jul 21;133(1):155–67. doi: 10.1007/s00702-025-02986-0 (PMC12827452; doi:10.1007/s00702-025-02986-0)
Supplement: Supplementary file 1 — Supplementary file1 (PDF 1981 KB) [file 702_2025_2986_MOESM1_ESM.pdf]

Supplementary Information

Table S1. Oligonucleotides for qRT-PCR.

Gene	Forward primer (5'-3')	Reverse primer (5'-3')	Manufacturer	Annealing Temp (^{0}C)	PCR product Melting point (^{0}C)	PCR Efficiency according to LinReg (%)
Rpl13a	GCCAGAAATGTTGATGCCTT	AGATGGCGGAGGTGCAG	Eurofins	56,5	87,5	98,5
Rpl19a	GTGGCAAGAAGAAGGTCTGG	GCCCATCTTTGATGAGCTTC	Eurofins	58,4	81,0	98,0
GAPDH	GAAGGTGAAGGTCGGAGT	GAAGATGGTGATGGGATTTC	Eurofins	55,7	82,0	95,0
Clock	CCAGCAGTTTCATGAGATGC	GAGGTCATTTCATAGCTGAGC	Eurofins	57,6	80,5	96,0
Bmal1	AAGGATGGCTGTTCAGCACATGA	CAAAAATCCATCTGCTGCCCTG	Eurofins	60,5	78,5	95,0
Per1	TGGGGACAACAGAACAGAGAA	AGGACACTCCTGCGACCA	Eurofins	58,1	85,0	99,5
Per2	GTATCCATTCATGCTGGGCT	TCGTTTGAACTGCGGTGAC	Eurofins	57,0	85,5	95,5
Per3	TCAGTGTTTGGTGGAAGGAA	TCTGGGTCAGCAGCTCTACA	Eurofins	57,4	81,5	98,5
Cry1	CACGAATCACAAACAGACGG	TACATCCTGGACCCCTGGT	Eurofins	58,1	82,0	99,0
Ctnnb1	According to manufacturer (Gene Globe ID QT00077882)	According to manufacturer (Gene Globe ID QT00077882)	Qiagen 249900	According to manufacturer (Gene Globe ID QT00077882)	79,0	100,0
Dkk1	According to manufacturer (Gene Globe ID QT00009093)	According to manufacturer (Gene Globe ID QT00009093)	Qiagen 249900	According to manufacturer (Gene Globe ID QT00077882)	77,5	99,5
Dkk3	According to manufacturer (Gene Globe ID QT00036057)	According to manufacturer (Gene Globe ID QT00036057)	Qiagen 249900	According to manufacturer (Gene Globe ID QT00077882)	87,5	99,0
Lrp6	According to manufacturer (Gene Globe ID QT00043176)	According to manufacturer (Gene Globe ID QT00043176)	Qiagen 249900	According to manufacturer (Gene Globe ID QT00077882)	80,0	99,5
Sirt1	According to manufacturer (Gene Globe ID QT00051261)	According to manufacturer (Gene Globe ID QT00051261)	Qiagen 249900	According to manufacturer (Gene Globe ID QT00077882)	76,5	100,0
Foxo1	According to manufacturer (Gene Globe ID QT00044247)	According to manufacturer (Gene Globe ID QT00044247)	Qiagen 249900	According to manufacturer (Gene Globe ID QT00077882)	78,0	100,0

Notes: Reference genes are marked in green.

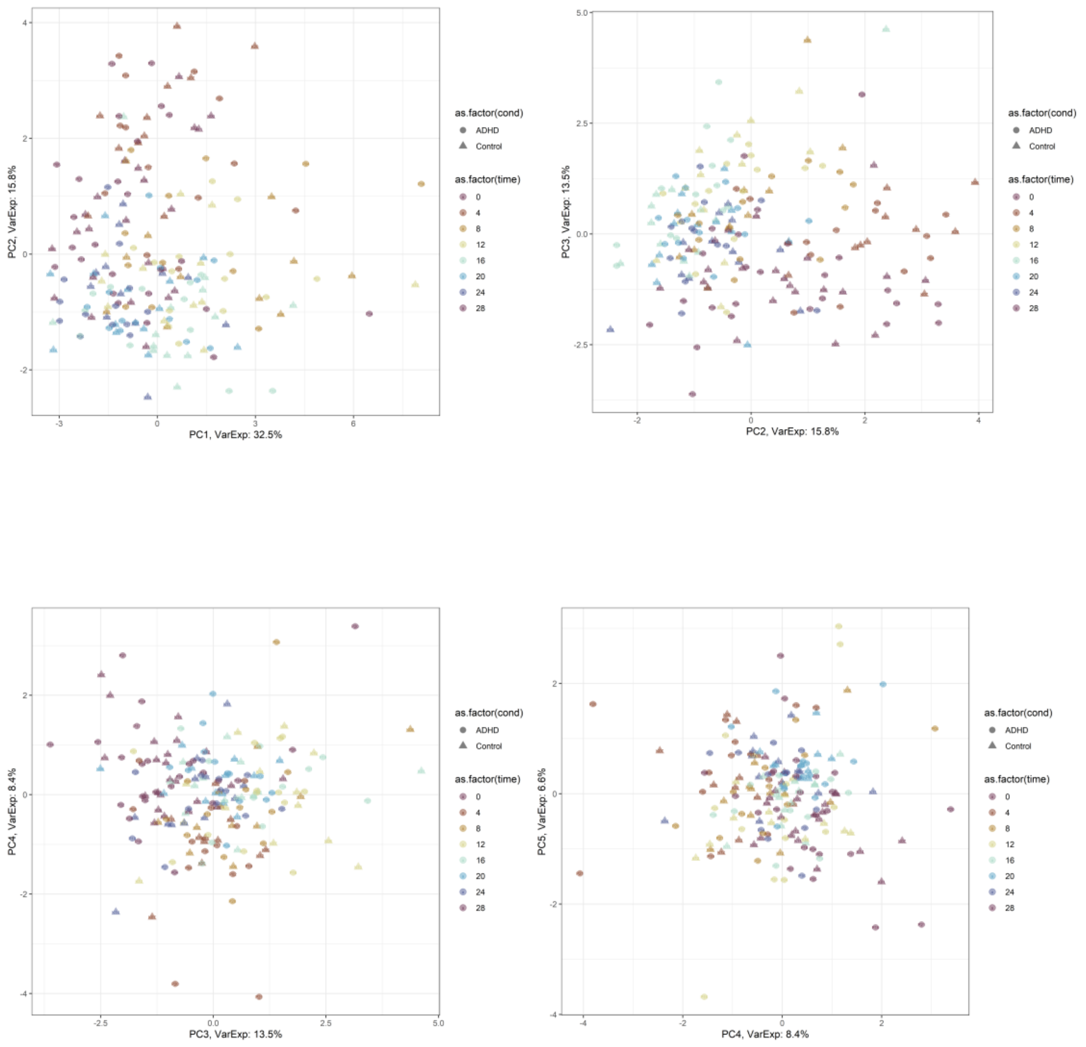

Figure S1. Principal Component Analysis (PCA) of gene expression data in Control and ADHD groups. PCA was conducted on the normalized expression profiles of all clock genes across time points. Scatter plots of consecutive PC pairs (PC1 vs PC2, PC2 vs PC3, etc.) are shown.

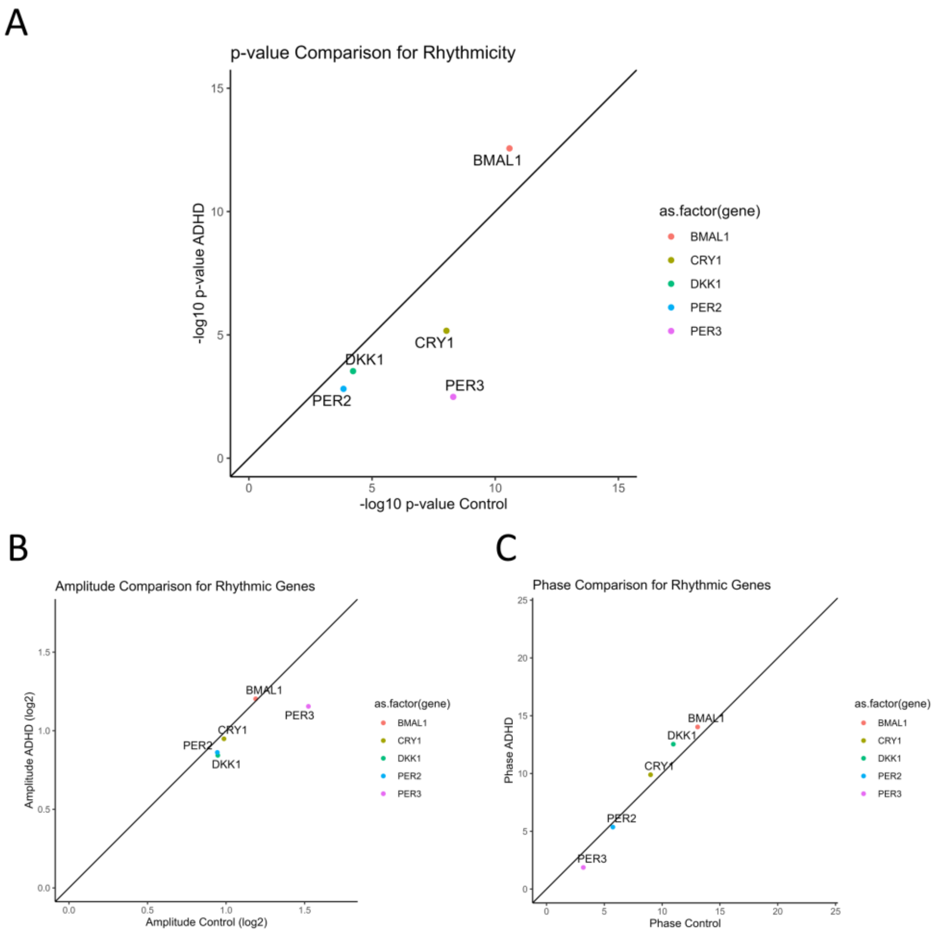

Figure S2. Rhythmicity of gene expression. A. The -log10(p-value) scatter plots of rhythmic gene expression between the control and ADHD groups. The results derive from the likelihood ratio test (LRT). If a gene falls above the line, it has stronger rhythmicity in ADHD. If a gene falls below the line, it has stronger rhythmicity in controls. **B.** Comparison of amplitude between the control and ADHD groups (log2). Points above the diagonal indicate a higher amplitude in ADHD. Points below the diagonal indicate a higher amplitude in controls. **C.** Comparison of phase timing between the control and ADHD groups. Points off the diagonal indicate a phase shift between the control and ADHD group.

Table S2. Group differences in rhythmicity parameters (Control vs. ADHD)

Gene	p_LRT	ChiSq	ΔMean ADHDvsControl	ΔCos ADHDvsControl	ΔSin ADHDvsControl	SE ΔMean	SE ΔCos	SE ΔSin
BMAL1	0.447	2.660	0.049	0.052	-0.140	0.069	0.094	0.099
CLOCK	0.726	1.314	0.014	0.034	-0.097	0.063	0.085	0.090
CTNNB1	0.887	0.639	0.017	0.016	-0.104	0.092	0.125	0.132
CRY1	0.708	1.388	0.002	-0.055	-0.100	0.072	0.098	0.103
DKK1	0.535	2.183	0.020	0.039	-0.186	0.089	0.120	0.127
DKK3	0.273	3.894	-0.022	-0.156	0.236	0.100	0.135	0.143
FOXO1	0.601	1.864	0.007	-0.096	0.231	0.126	0.170	0.180
LRP6	0.897	0.598	0.040	-0.095	0.058	0.108	0.146	0.155
PER2	0.977	0.202	-0.005	0.039	-0.046	0.093	0.126	0.133
PER3	0.485	2.446	0.012	-0.003	-0.293	0.131	0.177	0.188
PER1	0.815	0.944	0.063	-0.130	-0.068	0.119	0.162	0.171
SIRT1	0.734	1.279	-0.017	-0.141	0.122	0.119	0.161	0.170

Notes: Gene: gene name, p_LRT: p-value from likelihood ratio test, ChiSq: chi-square statistic, ΔMean ADHDvsControl: difference in baseline expression between ADHD and Control, ΔCos ADHDvsControl: difference in cosine component (rhythmicity shape/phase), ΔSin ADHDvsControl: difference in sine component (rhythmicity shape/phase), SE ΔMean: standard error of ΔMean, SE ΔCos: standard error of ΔCos, SE ΔSin: standard error of ΔSin.

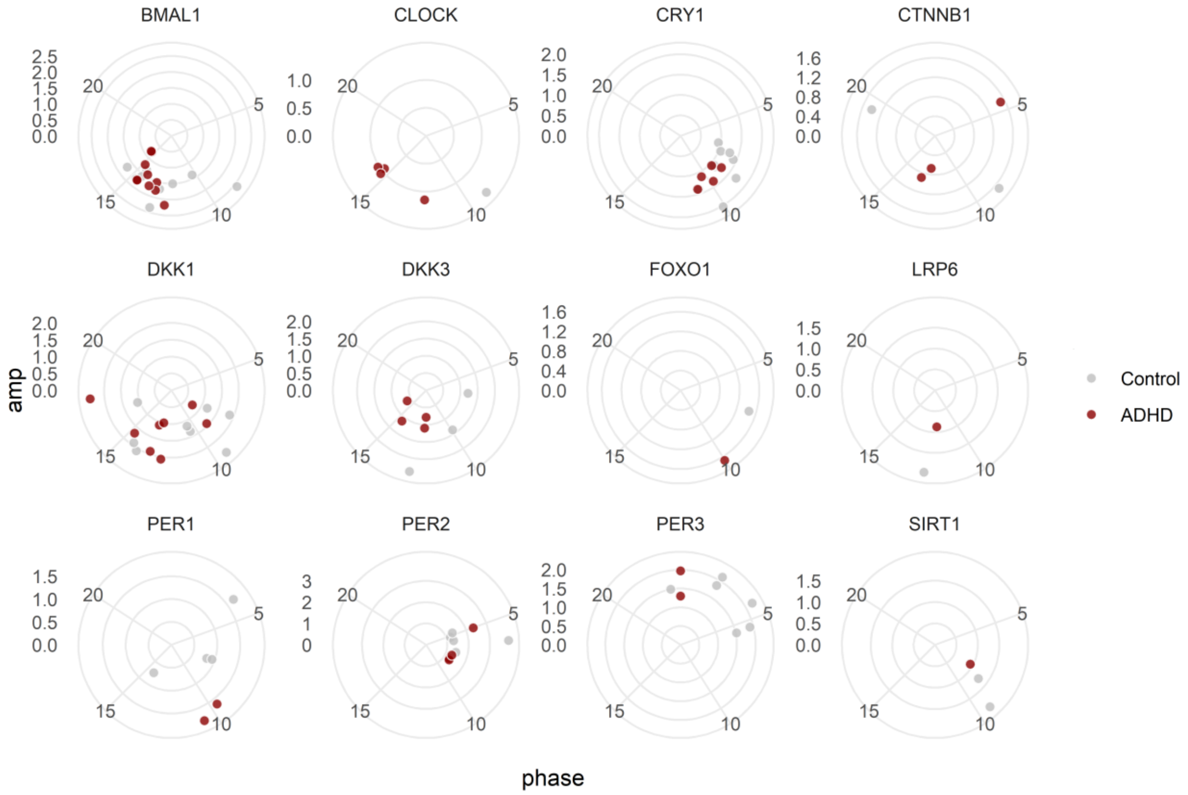

Figure S3. Individual-level rhythmicity. Each point represents the amplitude and phase of a rhythmic gene in individuals from the control (gray) or ADHD (dark red) group. The phase (x-axis in polar coordinates) represents the peak timing of gene expression within a 24-hour period, while the amplitude (y-axis) represents the strength of rhythmic oscillation. Only genes with significant rhythmicity are shown. A gene was considered rhythmic if the likelihood ratio test (LRT) in the harmonic regression was significant at a threshold of $p < 0.05$.

Table S3. Associations between clock and target genes for phase (unfiltered dataset)

ClockGene	TargetGene	Group	R2	R	p_value	n
BMAL1	*CTNNB1*	Control	0.021	0.147	0.633	13
BMAL1	*DKK1*	Control	0.005	0.069	0.823	13
BMAL1	*DKK3*	Control	0.075	0.273	0.366	13
BMAL1	*LRP6*	Control	0.004	-0.063	0.839	13
BMAL1	*SIRT1*	Control	0.033	0.182	0.553	13
BMAL1	*FOXO1*	Control	0.063	0.251	0.409	13
CLOCK	*CTNNB1*	Control	0.174	-0.418	0.156	13
CLOCK	*DKK1*	Control	0.167	-0.408	0.166	13
CLOCK	*DKK3*	Control	0.014	-0.117	0.703	13
CLOCK	*LRP6*	Control	0.475	-0.689	**0.009**	13
CLOCK	*SIRT1*	Control	0.020	-0.140	0.649	13
CLOCK	*FOXO1*	Control	0.010	0.098	0.749	13
CRY1	*CTNNB1*	Control	0.032	0.180	0.556	13
CRY1	*DKK1*	Control	0.004	-0.063	0.839	13
CRY1	*DKK3*	Control	0.165	0.406	0.168	13
CRY1	*LRP6*	Control	0.005	-0.070	0.819	13
CRY1	*SIRT1*	Control	0.011	-0.104	0.735	13
CRY1	*FOXO1*	Control	0.019	0.137	0.656	13
PER1	*CTNNB1*	Control	0.013	0.113	0.714	13
PER1	*DKK1*	Control	0.016	-0.127	0.679	13
PER1	*DKK3*	Control	0.338	0.581	**0.037**	13
PER1	*LRP6*	Control	0.038	-0.194	0.525	13
PER1	*SIRT1*	Control	0.001	0.030	0.922	13
PER1	*FOXO1*	Control	0.393	0.627	**0.022**	13
PER2	*CTNNB1*	Control	0.209	0.457	0.117	13
PER2	*DKK1*	Control	0.045	0.212	0.486	13
PER2	*DKK3*	Control	0.001	0.026	0.934	13
PER2	*LRP6*	Control	0.067	0.259	0.392	13
PER2	*SIRT1*	Control	0.046	0.214	0.483	13
PER2	*FOXO1*	Control	0.020	-0.140	0.649	13
PER3	*CTNNB1*	Control	0.001	0.026	0.934	13
PER3	*DKK1*	Control	0.000	-0.005	0.988	13
PER3	*DKK3*	Control	0.150	0.388	0.191	13
PER3	*LRP6*	Control	0.000	-0.015	0.962	13
PER3	*SIRT1*	Control	0.010	0.098	0.750	13
PER3	*FOXO1*	Control	0.067	-0.259	0.394	13
BMAL1	*CTNNB1*	ADHD	0.127	-0.357	0.231	13
BMAL1	*DKK1*	ADHD	0.080	-0.283	0.348	13
BMAL1	*DKK3*	ADHD	0.067	-0.260	0.391	13
BMAL1	*LRP6*	ADHD	0.146	-0.382	0.198	13
BMAL1	*SIRT1*	ADHD	0.174	0.417	0.156	13
BMAL1	*FOXO1*	ADHD	0.047	0.217	0.477	13
CLOCK	*CTNNB1*	ADHD	0.476	0.690	**0.009**	13
CLOCK	*DKK1*	ADHD	0.156	0.395	0.182	13

CLOCK	*DKK3*	ADHD	0.069	0.264	0.384	13
CLOCK	*LRP6*	ADHD	0.531	0.729	**0.005**	13
CLOCK	*SIRT1*	ADHD	0.000	-0.016	0.958	13
CLOCK	*FOXO1*	ADHD	0.129	-0.359	0.229	13
CRY1	*CTNNB1*	ADHD	0.075	-0.274	0.364	13
CRY1	*DKK1*	ADHD	0.012	-0.111	0.717	13
CRY1	*DKK3*	ADHD	0.008	0.091	0.769	13
CRY1	*LRP6*	ADHD	0.042	-0.206	0.500	13
CRY1	*SIRT1*	ADHD	0.546	0.739	**0.004**	13
CRY1	*FOXO1*	ADHD	0.061	0.246	0.418	13
PER1	*CTNNB1*	ADHD	0.122	0.349	0.243	13
PER1	*DKK1*	ADHD	0.006	-0.077	0.803	13
PER1	*DKK3*	ADHD	0.001	0.029	0.924	13
PER1	*LRP6*	ADHD	0.019	0.139	0.652	13
PER1	*SIRT1*	ADHD	0.027	-0.164	0.594	13
PER1	*FOXO1*	ADHD	0.001	0.038	0.903	13
PER2	*CTNNB1*	ADHD	0.009	-0.097	0.754	13
PER2	*DKK1*	ADHD	0.026	-0.162	0.596	13
PER2	*DKK3*	ADHD	0.006	-0.078	0.801	13
PER2	*LRP6*	ADHD	0.057	-0.239	0.431	13
PER2	*SIRT1*	ADHD	0.000	0.005	0.988	13
PER2	*FOXO1*	ADHD	0.008	0.089	0.772	13
PER3	*CTNNB1*	ADHD	0.048	0.218	0.474	13
PER3	*DKK1*	ADHD	0.153	-0.391	0.187	13
PER3	*DKK3*	ADHD	0.016	-0.125	0.685	13
PER3	*LRP6*	ADHD	0.018	0.132	0.666	13
PER3	*SIRT1*	ADHD	0.393	-0.627	**0.022**	13
PER3	*FOXO1*	ADHD	0.353	-0.594	**0.032**	13

Notes. R^2 = model fit, R = Pearson correlation, p = p-value from regression, n = number of paired observations.

Table S4. Associations between clock and target genes for phase (dataset filtered for rhythmicity)

**ClockGene**	**TargetGene**	**Condition**	**R2**	**R**	**p_value**	**n**
BMAL1	*DKK1*	Control	0.737	-0.858	0.142	4
CRY1	*DKK1*	Control	0.234	0.484	0.408	5
PER3	*DKK1*	Control	0.072	0.269	0.827	3
BMAL1	*DKK1*	ADHD	0.564	0.751	***0.085***	6
BMAL1	*DKK3*	ADHD	0.519	0.721	0.488	3
CLOCK	*DKK1*	ADHD	0.999	1.000	**0.020**	3
CRY1	*DKK1*	ADHD	0.011	-0.104	0.934	3
PER2	*DKK1*	ADHD	0.736	0.858	0.142	4

Notes. R^2 = model fit, R = Pearson correlation, p = p-value from regression, n = number of paired observations.

Table S5. Associations between clock and target genes for amplitude (unfiltered dataset)

ClockGene	TargetGene	Condition	R2	R	p_value	n
BMAL1	*CTNNB1*	Control	0.255	-0.505	***0.078***	13
BMAL1	*DKK1*	Control	0.177	-0.420	0.153	13
BMAL1	*DKK3*	Control	0.119	-0.345	0.248	13
BMAL1	*LRP6*	Control	0.416	-0.645	**0.017**	13
BMAL1	*SIRT1*	Control	0.250	-0.500	***0.082***	13
BMAL1	*FOXO1*	Control	0.420	-0.648	**0.017**	13
CLOCK	*CTNNB1*	Control	0.000	-0.001	0.997	13
CLOCK	*DKK1*	Control	0.104	0.323	0.282	13
CLOCK	*DKK3*	Control	0.007	-0.083	0.787	13
CLOCK	*LRP6*	Control	0.020	-0.140	0.647	13
CLOCK	*SIRT1*	Control	0.021	0.145	0.637	13
CLOCK	*FOXO1*	Control	0.109	-0.330	0.271	13
CRY1	*CTNNB1*	Control	0.000	-0.014	0.963	13
CRY1	*DKK1*	Control	0.138	0.371	0.212	13
CRY1	*DKK3*	Control	0.177	0.420	0.153	13
CRY1	*LRP6*	Control	0.009	-0.094	0.760	13
CRY1	*SIRT1*	Control	0.004	-0.064	0.835	13
CRY1	*FOXO1*	Control	0.114	0.338	0.259	13
PER1	*CTNNB1*	Control	0.016	-0.126	0.682	13
PER1	*DKK1*	Control	0.105	0.324	0.280	13
PER1	*DKK3*	Control	0.013	-0.114	0.711	13
PER1	*LRP6*	Control	0.058	-0.240	0.429	13
PER1	*SIRT1*	Control	0.045	-0.212	0.486	13
PER1	*FOXO1*	Control	0.150	-0.387	0.191	13
PER2	*CTNNB1*	Control	0.036	-0.191	0.533	13
PER2	*DKK1*	Control	0.014	-0.117	0.704	13
PER2	*DKK3*	Control	0.305	-0.552	***0.050***	13
PER2	*LRP6*	Control	0.067	-0.258	0.395	13
PER2	*SIRT1*	Control	0.001	0.023	0.940	13
PER2	*FOXO1*	Control	0.114	-0.338	0.259	13
PER3	*CTNNB1*	Control	0.026	-0.163	0.595	13
PER3	*DKK1*	Control	0.004	-0.064	0.836	13
PER3	*DKK3*	Control	0.184	-0.428	0.144	13
PER3	*LRP6*	Control	0.050	-0.224	0.463	13
PER3	*SIRT1*	Control	0.106	-0.326	0.277	13
PER3	*FOXO1*	Control	0.006	-0.078	0.799	13
BMAL1	*CTNNB1*	ADHD	0.087	-0.295	0.327	13
BMAL1	*DKK1*	ADHD	0.033	-0.181	0.555	13
BMAL1	*DKK3*	ADHD	0.000	-0.011	0.972	13
BMAL1	*LRP6*	ADHD	0.004	-0.065	0.832	13
BMAL1	*SIRT1*	ADHD	0.152	0.390	0.188	13
BMAL1	*FOXO1*	ADHD	0.137	0.370	0.214	13
CLOCK	*CTNNB1*	ADHD	0.187	-0.433	0.140	13
CLOCK	*DKK1*	ADHD	0.062	-0.248	0.413	13

CLOCK	*DKK3*	ADHD	0.189	-0.434	0.138	13
CLOCK	*LRP6*	ADHD	0.045	0.212	0.487	13
CLOCK	*SIRT1*	ADHD	0.002	-0.042	0.892	13
CLOCK	*FOXO1*	ADHD	0.063	0.250	0.409	13
CRY1	*CTNNB1*	ADHD	0.008	0.090	0.771	13
CRY1	*DKK1*	ADHD	0.091	0.302	0.316	13
CRY1	*DKK3*	ADHD	0.090	0.299	0.321	13
CRY1	*LRP6*	ADHD	0.005	-0.073	0.812	13
CRY1	*SIRT1*	ADHD	0.026	0.161	0.599	13
CRY1	*FOXO1*	ADHD	0.004	-0.067	0.828	13
PER1	*CTNNB1*	ADHD	0.013	0.115	0.708	13
PER1	*DKK1*	ADHD	0.077	-0.278	0.358	13
PER1	*DKK3*	ADHD	0.004	-0.059	0.848	13
PER1	*LRP6*	ADHD	0.072	0.269	0.375	13
PER1	*SIRT1*	ADHD	0.123	0.351	0.239	13
PER1	*FOXO1*	ADHD	0.027	0.164	0.592	13
PER2	*CTNNB1*	ADHD	0.000	0.014	0.964	13
PER2	*DKK1*	ADHD	0.001	0.024	0.937	13
PER2	*DKK3*	ADHD	0.037	0.192	0.529	13
PER2	*LRP6*	ADHD	0.304	0.551	***0.051***	13
PER2	*SIRT1*	ADHD	0.062	0.248	0.414	13
PER2	*FOXO1*	ADHD	0.115	0.340	0.256	13
PER3	*CTNNB1*	ADHD	0.094	-0.306	0.309	13
PER3	*DKK1*	ADHD	0.450	-0.671	**0.012**	13
PER3	*DKK3*	ADHD	0.138	-0.372	0.211	13
PER3	*LRP6*	ADHD	0.011	-0.103	0.738	13
PER3	*SIRT1*	ADHD	0.001	-0.039	0.900	13
PER3	*FOXO1*	ADHD	0.000	0.003	0.992	13

Notes. R^2 = model fit, R = Pearson correlation, p = p-value from regression, n = number of paired observations.

Table S6. Associations between clock and target genes for amplitude (dataset filtered for rhythmicity)

**ClockGene**	**TargetGene**	**Condition**	**R2**	**R**	**p_value**	**n**
BMAL1	*DKK1*	Control	0.921	0.960	**0.040**	4
CRY1	*DKK1*	Control	0.180	-0.425	0.476	5
PER3	*DKK1*	Control	0.598	0.774	0.437	3
BMAL1	*DKK1*	ADHD	0.036	-0.190	0.718	6
BMAL1	*DKK3*	ADHD	0.833	0.912	0.268	3
CLOCK	*DKK1*	ADHD	0.011	-0.106	0.932	3
CRY1	*DKK1*	ADHD	0.990	0.995	0.065	3
PER2	*DKK1*	ADHD	0.509	-0.714	0.286	4

Notes. R^2 = model fit, R = Pearson correlation, p = p-value from regression, n = number of paired observations.

Table S7. Rayleigh test statistics for circadian phase clustering by gene and group

Gene	Group	Mean phase	Rayleigh p	r statistics	n
BMAL1	Control	13.012	**<0.001**	0.752	13
BMAL1	ADHD	14.320	**<0.001**	0.852	13
CLOCK	Control	10.085	***0.053***	0.471	13
CLOCK	ADHD	10.788	0.174	0.368	13
CTNNB1	Control	9.881	0.163	0.374	13
CTNNB1	ADHD	10.732	***0.060***	0.462	13
CRY1	Control	9.227	**<0.001**	0.861	13
CRY1	ADHD	9.846	**<0.001**	0.808	13
DKK1	Control	11.207	**0.006**	0.604	13
DKK1	ADHD	11.395	**0.006**	0.611	13
DKK3	Control	16.736	0.466	0.245	13
DKK3	ADHD	10.405	**0.002**	0.655	13
FOXO1	Control	8.551	**0.025**	0.524	13
FOXO1	ADHD	9.091	**<0.001**	0.859	13
LRP6	Control	11.903	0.510	0.231	13
LRP6	ADHD	13.599	0.473	0.243	13
PER1	Control	7.655	**0.006**	0.610	13
PER1	ADHD	7.780	**0.009**	0.588	13
PER2	Control	6.296	**0.001**	0.693	13
PER2	ADHD	5.342	**<0.001**	0.766	13
PER3	Control	3.128	**<0.001**	0.880	13
PER3	ADHD	2.594	**<0.001**	0.836	13
SIRT1	Control	9.782	0.542	0.220	13
SIRT1	ADHD	10.740	***0.072***	0.447	13

Table S8. Circular–circular correlations between clock and WNT/stress genes by group

ClockGene	TargetGene	Condition	rho	P value	n
BMAL1	*CTNNB1*	Control	-0.261	0.794	13
BMAL1	*DKK1*	Control	-0.226	0.821	13
BMAL1	*DKK3*	Control	1.320	0.187	13
BMAL1	*LRP6*	Control	-0.321	0.748	13
BMAL1	*SIRT1*	Control	0.348	0.728	13
BMAL1	*FOXO1*	Control	0.379	0.705	13
CLOCK	*CTNNB1*	Control	-0.639	0.523	13
CLOCK	*DKK1*	Control	-1.453	0.146	13
CLOCK	*DKK3*	Control	0.214	0.831	13
CLOCK	*LRP6*	Control	-1.559	0.119	13
CLOCK	*SIRT1*	Control	0.270	0.787	13
CLOCK	*FOXO1*	Control	0.276	0.783	13
CRY1	*CTNNB1*	Control	0.209	0.834	13
CRY1	*DKK1*	Control	-0.158	0.874	13
CRY1	*DKK3*	Control	1.098	0.272	13
CRY1	*LRP6*	Control	-0.249	0.803	13

CRY1	*SIRT1*	Control	-0.351	0.725	13
CRY1	*FOXO1*	Control	2.152	**0.031**	13
PER1	*CTNNB1*	Control	0.035	0.972	13
PER1	*DKK1*	Control	-1.182	0.237	13
PER1	*DKK3*	Control	0.840	0.401	13
PER1	*LRP6*	Control	-0.849	0.396	13
PER1	*SIRT1*	Control	-1.522	0.128	13
PER1	*FOXO1*	Control	1.451	0.147	13
PER2	*CTNNB1*	Control	1.519	0.129	13
PER2	*DKK1*	Control	0.278	0.781	13
PER2	*DKK3*	Control	-0.267	0.790	13
PER2	*LRP6*	Control	0.114	0.909	13
PER2	*SIRT1*	Control	-0.538	0.590	13
PER2	*FOXO1*	Control	1.371	0.170	13
PER3	*CTNNB1*	Control	0.387	0.699	13
PER3	*DKK1*	Control	0.435	0.664	13
PER3	*DKK3*	Control	-1.021	0.307	13
PER3	*LRP6*	Control	0.209	0.835	13
PER3	*SIRT1*	Control	0.107	0.914	13
PER3	*FOXO1*	Control	0.605	0.545	13
BMAL1	*CTNNB1*	ADHD	-0.889	0.374	13
BMAL1	*DKK1*	ADHD	-0.851	0.395	13
BMAL1	*DKK3*	ADHD	-1.146	0.252	13
BMAL1	*LRP6*	ADHD	-0.790	0.430	13
BMAL1	*SIRT1*	ADHD	1.020	0.308	13
BMAL1	*FOXO1*	ADHD	1.194	0.232	13
CLOCK	*CTNNB1*	ADHD	2.179	**0.029**	13
CLOCK	*DKK1*	ADHD	0.796	0.426	13
CLOCK	*DKK3*	ADHD	0.584	0.559	13
CLOCK	*LRP6*	ADHD	1.831	***0.067***	13
CLOCK	*SIRT1*	ADHD	0.239	0.811	13
CLOCK	*FOXO1*	ADHD	-1.272	0.204	13
CRY1	*CTNNB1*	ADHD	0.612	0.541	13
CRY1	*DKK1*	ADHD	1.400	0.161	13
CRY1	*DKK3*	ADHD	1.498	0.134	13
CRY1	*LRP6*	ADHD	0.100	0.920	13
CRY1	*SIRT1*	ADHD	3.118	**0.002**	13
CRY1	*FOXO1*	ADHD	1.236	0.217	13
PER1	*CTNNB1*	ADHD	0.078	0.937	13
PER1	*DKK1*	ADHD	-1.036	0.300	13
PER1	*DKK3*	ADHD	-1.668	***0.095***	13
PER1	*LRP6*	ADHD	0.037	0.970	13
PER1	*SIRT1*	ADHD	-2.306	**0.021**	13
PER1	*FOXO1*	ADHD	-1.133	0.257	13
PER2	*CTNNB1*	ADHD	1.039	0.299	13
PER2	*DKK1*	ADHD	1.506	0.132	13
PER2	*DKK3*	ADHD	1.047	0.295	13
PER2	*LRP6*	ADHD	0.461	0.645	13

PER2	*SIRT1*	ADHD	2.121	**0.034**	13
PER2	*FOXO1*	ADHD	2.733	**0.006**	13
PER3	*CTNNB1*	ADHD	-0.949	0.343	13
PER3	*DKK1*	ADHD	0.266	0.790	13
PER3	*DKK3*	ADHD	-0.250	0.802	13
PER3	*LRP6*	ADHD	-1.117	0.264	13
PER3	*SIRT1*	ADHD	1.595	0.111	13
PER3	*FOXO1*	ADHD	2.097	**0.036**	13

Notes. rho = circular correlation coefficient; p = p-value; n = number of individuals per group.

Table S9. Spearman correlations between rhythmic parameters of gene expression and clinical/sleep quality data

**Gene parameter**	**Score**	**r**	**p**	**n**	**FDR**
BMAL1_amp	CAARS	0.867	**0.005**	9	0.108
CLOCK_amp	CAARS	0.383	0.313	9	0.682
CTNNB1_amp	CAARS	-0.050	0.912	9	1.000
CRY1_amp	CAARS	0.283	0.463	9	0.855
DKK1_amp	CAARS	0.000	1.000	9	1.000
DKK3_amp	CAARS	0.383	0.313	9	0.682
FOXO1_amp	CAARS	-0.033	0.948	9	1.000
LRP6_amp	CAARS	0.350	0.359	9	0.717
PER2_amp	CAARS	-0.450	0.230	9	0.613
PER3_amp	CAARS	0.450	0.230	9	0.613
PER1_amp	CAARS	0.800	**0.014**	9	0.166
SIRT1_amp	CAARS	0.117	0.776	9	1.000
BMAL1_phase	CAARS	-0.500	0.178	9	0.613
CLOCK_phase	CAARS	0.250	0.521	9	0.893
CTNNB1_phase	CAARS	0.200	0.613	9	0.981
CRY1_phase	CAARS	-0.650	0.067	9	0.399
DKK1_phase	CAARS	0.067	0.880	9	1.000
DKK3_phase	CAARS	0.167	0.678	9	1.000
FOXO1_phase	CAARS	-0.150	0.708	9	1.000
LRP6_phase	CAARS	0.017	0.982	9	1.000
PER2_phase	CAARS	0.100	0.810	9	1.000
PER3_phase	CAARS	0.717	**0.037**	9	0.295
PER1_phase	CAARS	0.483	0.194	9	0.613
SIRT1_phase	CAARS	-0.450	0.230	9	0.613
BMAL1_amp	PSQI	0.228	0.555	9	0.889
CLOCK_amp	PSQI	-0.051	0.897	9	0.972
CTNNB1_amp	PSQI	-0.489	0.181	9	0.621
CRY1_amp	PSQI	-0.591	0.094	9	0.621
DKK1_amp	PSQI	-0.464	0.208	9	0.624
DKK3_amp	PSQI	-0.506	0.164	9	0.621
FOXO1_amp	PSQI	-0.262	0.496	9	0.889
LRP6_amp	PSQI	0.008	0.983	9	0.983

PER2_amp	PSQI	0.042	0.914	9	0.972
PER3_amp	PSQI	0.034	0.931	9	0.972
PER1_amp	PSQI	0.101	0.795	9	0.972
SIRT1_amp	PSQI	-0.135	0.729	9	0.972
BMAL1_phase	PSQI	0.591	0.094	9	0.621
CLOCK_phase	PSQI	-0.549	0.126	9	0.621
CTNNB1_phase	PSQI	-0.405	0.279	9	0.745
CRY1_phase	PSQI	-0.498	0.173	9	0.621
DKK1_phase	PSQI	-0.321	0.400	9	0.873
DKK3_phase	PSQI	-0.076	0.846	9	0.972
FOXO1_phase	PSQI	0.160	0.680	9	0.972
LRP6_phase	PSQI	-0.245	0.526	9	0.889
PER2_phase	PSQI	-0.118	0.762	9	0.972
PER3_phase	PSQI	0.743	**0.022**	9	0.525
PER1_phase	PSQI	0.346	0.362	9	0.868
SIRT1_phase	PSQI	-0.279	0.468	9	0.889

Notes. CAARS, Conners' Adult ADHD Rating Scales; FDR, False Discovery Rate (correction per 24 tests within the instrument); gene parameter = amplitude, phase; n = number of individuals per group; p = p-value; PSQI, Pittsburgh Sleep Quality Index.

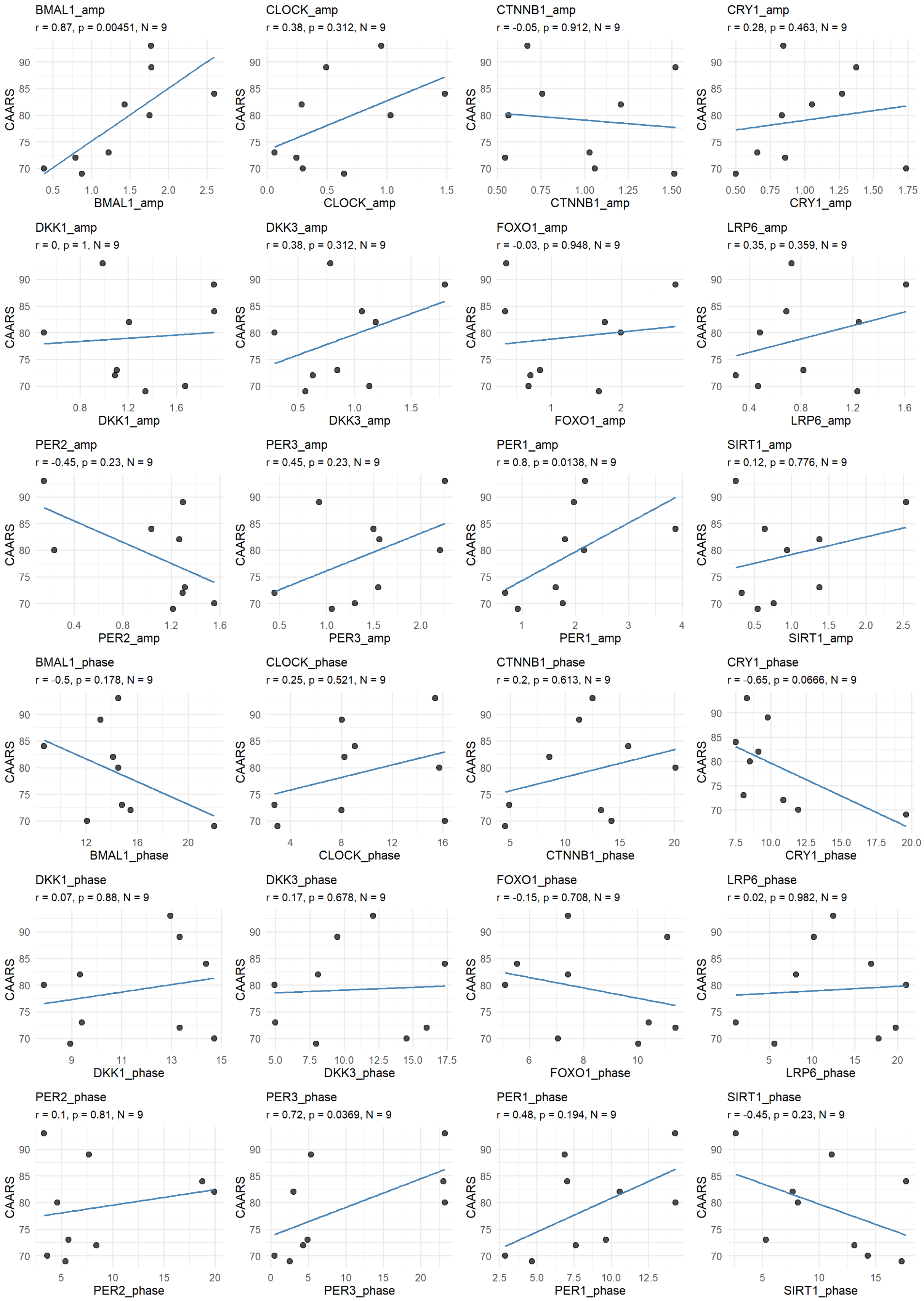

Figure S4. Scatterplots showing Spearman's correlations between gene expression parameters (amplitude and phase) and Conners' Adult ADHD Rating Scales CAARS scores.

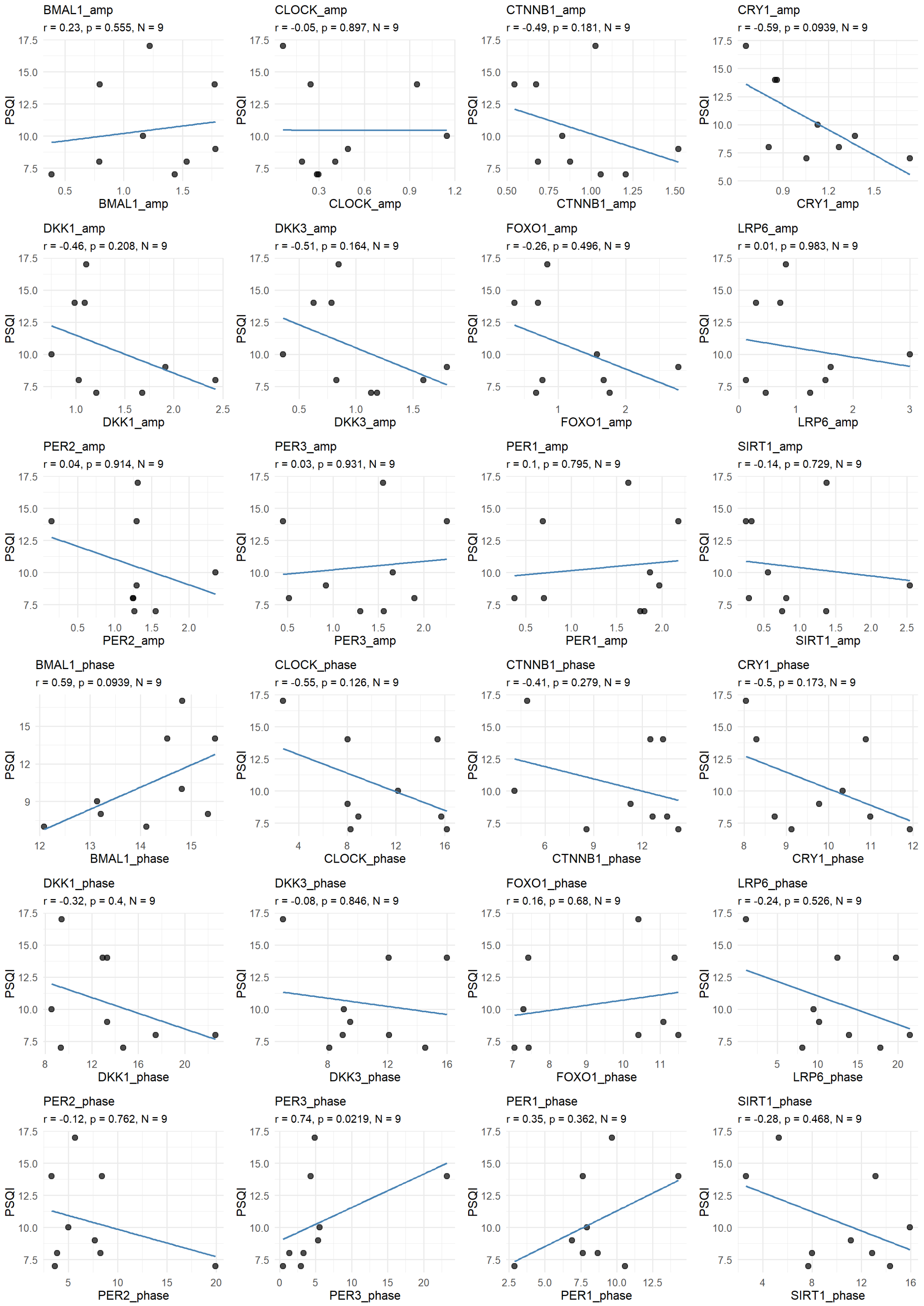

Figure S5. Scatterplots showing Spearman's correlations between gene expression parameters (amplitude and phase) and Pittsburgh Sleep Quality Index (PSQI) scores.
